# Imaging chromatin nanostructure with binding-activated localization microscopy based on DNA structure fluctuations

**DOI:** 10.1093/nar/gkw1301

**Published:** 2017-01-13

**Authors:** Aleksander Szczurek, Ludger Klewes, Jun Xing, Amine Gourram, Udo Birk, Hans Knecht, Jurek W. Dobrucki, Sabine Mai, Christoph Cremer

**Affiliations:** 1 Institute of Molecular Biology, 55128 Mainz, Germany; 2 University of Manitoba, Cancer Care Manitoba, Winnipeg R3E 0V9, Canada; 3 Physics Department University Mainz (JGU), 55128 Mainz, Germany; 4 Département de Médecine, CHUS, Université de Sherbrooke, 3001-12e Avenue Nord, Sherbrooke, Québec J1H 5N4, Canada; 5 Department of Medicine, Jewish General Hospital, McGill University, 3755 Côte-Ste-Catherine Road, Montreal, Québec H3T 1E2, Canada; 6 Department of Cell Biophysics, Faculty of Biochemistry, Biophysics and Biotechnology, Jagiellonian University, Kraków, Poland; 7 Kirchhoff Institute of Physics (KIP), and Institute of Pharmacy & Molecular Biotechnology (IPMB), University Heidelberg, Germany

## Abstract

Advanced light microscopy is an important tool for nanostructure analysis of chromatin. In this report we present a general concept for Single Molecule localization Microscopy (SMLM) super-resolved imaging of DNA-binding dyes based on modifying the properties of DNA and the dye. By careful adjustment of the chemical environment leading to local, reversible DNA melting and hybridization control over the fluorescence signal of the DNA-binding dye molecules can be introduced. We postulate a transient binding as the basis for our variation of binding-activated localization microscopy (BALM). We demonstrate that several intercalating and minor-groove binding DNA dyes can be used to register (optically isolate) only a few DNA-binding dye signals at a time. To highlight this DNA structure fluctuation-assisted BALM (fBALM), we applied it to measure, for the first time, nanoscale differences in nuclear architecture in model ischemia with an anticipated structural resolution of approximately 50 nm. Our data suggest that this approach may open an avenue for the enhanced microscopic analysis of chromatin nano-architecture and hence the microscopic analysis of nuclear structure aberrations occurring in various pathological conditions. It may also become possible to analyse nuclear nanostructure differences in different cell types, stages of development or environmental stress conditions.

## INTRODUCTION

In spite of a great deal of knowledge already available, the precise higher order spatial organisation of chromatin at the nanoscale is still a subject of an ongoing debate (1–3). Currently, the most plausible models are based on optical microscopy data and suggest the existence of small chromatin domains (4), or on chromosome conformation capture studies suggesting the existence of regions denoted as ‘topologically associated chromatin domains’ (TADs) (5,6). These small chromatin domains ranging in size from hundreds of thousands to millions of base pairs are believed to constitute a basic higher order chromatin organisation unit above the level of the individual nucleosomes, as for instance in DNA replication (7).

Among various techniques that contributed significantly to our current understanding of the genome organisation (reviewed in (3,8–10)), the recently emerged methods of super-resolution microscopy deserve a special attention (for review see (11). These methods have already been successfully applied to studies of the cell nucleus *in situ*. For instance, it was possible to demonstrate changes in chromatin organisation as well as in nuclear lamina and nuclear pore complex formation during bovine embryo pre-implantation development (12,13), dynamic structural rearrangements during human myelopoiesis (14), formation of previously unknown histone clutches varying in size depending on cell pluripotency (15), and chromatin condensation changes during model ischemia (16). Furthermore, using *in situ* hybridization distinct chromatin packaging for different epigenetic states at kilobase-to-megabase genomic length was observed (17). Such structural details may provide a solid background for the interpretation of other chromatin investigation methodologies, in particular molecular biology methods mostly requiring the information to be averaged over millions of cells.

For the highest resolution imaging of DNA-binding dyes, single molecule localization microscopy (SMLM, reviewed in (18)) has been applied in several ways, using different dyes and chemical environments (imaging buffers). For instance, photoconversion of DNA-bound Hoechst dyes in a glycerol based buffer devoid of oxygen allowed separation of single molecule signals based on modified spectral characteristics (19). In other approaches, cyanine double-helix intercalators YOYO-1, YO-PRO-1 or TOTO-3 dyes were visualized by using an imaging buffer devoid of oxygen and containing β-mercaptoethylamine (MEA) (20,21). Live-cell SMLM using minor-groove binding dye PicoGreen in the presence of 1 mM ascorbic acid and in the absence of oxygen was also reported (22). An approach termed ‘Binding Activated localization Microscopy’ (BALM) relied on a transient binding of YOYO-1 and PicoGreen to the DNA in a specially designed buffer containing millimolar concentrations of methyl viologen and ascorbic acid, with simultaneous oxygen deprivation (23).

Conceptually, BALM has evolved from the differential imaging of bound vs. unbound fluorescent dye molecules in ‘point accumulation for imaging in nanoscale topography’ (PAINT) (24). In BALM, once a fluorescent dye binds to DNA, it may repeatedly cycle through absorption and fluorescence emission until it is either released from the DNA or irreversibly bleached. Therefore, the DNA-bound dye molecule appears in the images as a bright ‘spot’ that can be precisely localized in space using SMLM principles (25). In contrast, freely diffusing molecules in the solvent remain essentially non-fluorescent and hence undetectable. Understandably, in BALM it is of utmost importance to accelerate the dynamic interaction of the dye with the DNA. Until now DNA–BALM was applied to isolated DNA threads or to bacterial genomes (23), i.e. to structures which are ∼3 orders of magnitude smaller in size than the mammalian cell nucleus.

In this study, we investigated the influence of various chemical environments affecting the structural stability of DNA stained with DNA-binding dyes in order to gain an insight into the mechanism underlying DNA–BALM. In addition, we evaluated the influence of the chemical environment on the preservation of nuclear structure. Utilizing these findings, we successfully applied, for the first time, our modified BALM approach to super-resolution imaging of whole mouse and human cell nuclei. This new methodological variety of SMLM termed by us *DNA structure fluctuation-assisted Binding Activated localization Microscopy* (further abbreviated fBALM) yielded a structure-based resolution of few tens of nanometer, and a Fourier Ring Correlation based structural resolution of 37 nm. We demonstrate quantitatively that our method performs well enough to resolve nano-scale changes of chromatin condensation reported before for ischemic conditions. In this report we also communicate that the nuclear DNA arrangement varies notably in different cancer cell lines.

## MATERIALS AND METHODS

### Confocal microscopy

**Figure 1.**
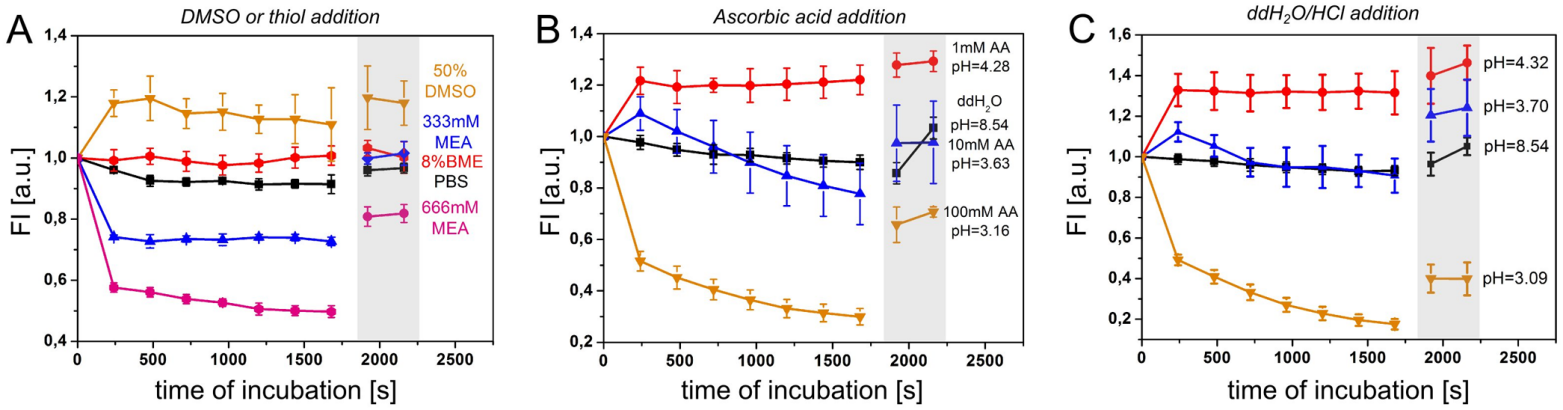
Influence of the chemical environment on YOYO-1 fluorescence signal intensity in nuclei of HeLa cells. Time course confocal microscopy of YOYO-1 was carried out after addition of 50% DMSO, 8% β-mercaptoethanol (BME) or 333 and 666 mM β-mercaptoethylamine (MEA) in PBS (**A**), 1 mM, 10 mM or 100 mM ascorbic acid (AA) in water (**B**) or H_2_O/HCl buffers with pH ranging from 8.54 to 3.09 (**C**). The first data-point corresponds to the fluorescence signal before addition of a buffer. Light-gray rectangle denotes data-points acquired after four and eight washing steps with either PBS (A) or H_2_O (B, C). Each curve represents measurements in >100 cells, in five to six fields of view. Error bars correspond to a standard deviation. FL: average fluorescence intensity per cell (arbitrary units).

For confocal microscopy measurements HeLa cells were seeded onto 500 μl μ-slide eight-well chambers (IBIDI, Germany). Next, they were fixed (3.7% formaldehyde, 15 min), Triton X-100 treated (0.5%, 15 min). And RNase treated (37°C, overnight). For DNA-association assay, the cells inside the chamber were placed in the focal plane of a confocal microscope (Leica SP5 equipped with 63×, 1.4 NA objective, 488 nm argon laser line and photon counting detector), an initial (background) image was taken and subsequently 250 μl of twice concentrated dye solution was added to 250 μl PBS within a well. Images were acquired every 2 min and the average fluorescence signal of DNA-bound dye was quantified. For studies on DNA structure fluctuations (Figure 1, Supplementary Figures S2 and S3) pre-stained cells were used (1 h/12 nM YOYO-1, 40 min/1:10 000 PicoGreen, or 40 min/30 nM YO-PRO-1), an initial image was taken in H_2_O or PBS, followed by a buffer exchange to one of the following buffers containing: hydrochloric acid, ascorbic acid, β-mercaptoethanol, β-mercaptoethylamine (Sigma-Aldrich, Germany) or DMSO. Signal was traced over 28 min every 4 min, followed by four washing steps with 750 μl of H_2_O or PBS and image acquisition, carried out twice. In order to measure a change in chromatin structure, cells were labeled with 10 μM 5-ethynyl-2΄-deoxyuridine (EdU) for 12 min and stained with Alexa 555 once fixed. 3D confocal images were acquired prior to and after exchanging the chemical environment. Nearest neighbor distances and cell volumes were calculated using Matlab.

### Single molecule localization microscopy

HL-1 murine cardiac muscle cells (26) were cultured *in vitro*, fixed, and treated with RNA as described in (16). For cells labeled with YOYO-1 (12 nM, 45 min), the following fBALM imaging cocktail was used: 0.5 mg/ml glucose oxidase (GOX), 40 μg/ml catalase (CAT) and 10% (w/v) glucose, all in PBS. Histone H3 was immuno-labeled with a secondary Alexa 647-antibody (imaged in GOX/CAT/glucose buffer with 50 mM cysteamine) whereas EdU was labeled with Alexa 488 (imaged in ProlongGold^®^, Lifetechnologies). In order to investigate chromatin condensation in ischemia (hypoglycaemia + hypoxia), we used a previously developed preparation protocol (16). Hodgkin's lymphoma cell line HDLM-2 was prepared for microscopy as previously reported with minor modifications (27). Neuroblastoma Neuro-2a cells (ATCC^®^ CL-131™) were cultured in Dulbecco's modified Eagle medium with glutamine, nonessential amino acids, and 10% fetal bovine serum (Gibco, Germany). For fBALM and Alexa 488 imaging we used 1–2 kW/cm^2^ 491 nm laser intensity (Cobolt), 30 000, 20 ms and <5000, 50 ms frames acquired, respectively. For Alexa 647 we used ∼2 kW/cm^2^ 647 nm laser (Omicron) and 25 000 frames with 25 ms camera exposure. Signal was collected with a 63×, 1.4 NA objective and detected using 12 bit CCD camera (PCO imaging). For more details see (19). Data were analysed using fastSPDM software running in MATLAB (28) as previously described (16). Briefly, the obtained list of localizations (signals in raw images with peak-intensity exceeding 3x noise standard deviation above background) was filtered with respect to the signal diameter (∼30% broadest ones filtered out), localizations appearing in subsequent frames closer than 2× average localization precision were combined. Fourier ring correlation (FRC) analysis was performed utilising a custom written MATLAB program similar to the method described in (29,30). In order to compare DNA distribution in fBALM images we binned single molecule data in 60 × 60 nm^2^ grid and plotted histograms with respect to a number of signals per grid-square. For visualization of the data we used an approach similar to triangulation (31). Here, triangles were replaced with ‘Wigner-Seitz’/Voronoi ‘cells’. Final reconstructions have a pixel size corresponding to 5 nm.

For more detailed information, see extended materials and methods in supplementary materials.

## RESULTS

### Chemical environment influences YOYO-1 signal intensity over time

Binding activated localization microscopy as proposed originally (23) relies on the presence of a DNA-binding dye in the imaging medium of a composition designed to facilitate the dynamic interaction with the DNA and promote fluorescence emission. While this approach is successful for small and easily accessible structures, it poses a major challenge to the nanoscale imaging of the relatively large, highly structured and membrane enclosed eukaryotic cell nucleus. Due to the limited diffusion, labelling with DNA-binding dyes begins at the nuclear periphery and progresses into the inner parts of the cell nucleus over 60 min (dye penetration artifacts are discussed in Supplementary Figure S1 and Supplementary Note 3).

In the original BALM approach (23), YOYO-1, a cyanine homodimer which exhibits optimal fluorogenic features (for details see Supplementary Table S1) was used. When YOYO-1 was introduced into the imaging medium, the dye molecules released from DNA (or bleached out) were constantly replenished by the molecules from the surrounding buffer. This rapid exchange is possible when a pool of unbleached dye molecules is present in a close proximity to the imaged structure. To fulfill this requirement in imaging the whole cell nuclei, we designed a method in which fixed cells are first preloaded with YOYO-1 for as long as it is required to saturate the binding sites throughout the entire cell nucleus. The abundant freely diffusing YOYO-1 is washed out and subsequently the chemical conditions are changed in order to induce DNA structural fluctuations affecting the signal intensity of YOYO-1. In case of YOYO-1 being released from the DNA, we expected that it would stay inside the cell nucleus for tens of minutes because of the constrained diffusion (Supplementary Figure S1). If these assumptions are valid, the concentration of the unbound YOYO-1 molecules within the cell nucleus should facilitate high-density BALM on DNA, while the peripheral artifacts should be minimised (Supplementary Figure S1B).

YOYO-1 is known to require a stable DNA double helix for intercalation and fluorescence emission (32). By targeting the hydrogen bonds responsible for maintaining of double-stranded DNA (dsDNA) it should be possible to affect the local stability of the DNA–dye complex. This was already demonstrated in a DNA bar-code analysis of single DNA fibres where YOYO-1 signal dropped to a background level upon sequence specific denaturation (33). Therefore, utilizing time-lapse confocal microscopy of the cells labeled with YOYO-1, we evaluated factors known to influence dsDNA stability, including DMSO (34) and low pH (35,36). We also examined the influence of ascorbic acid and primary thiols formerly used in SMLM of DNA-binding dyes (20,22,23) (Figure 1). In our assay we relied on the fact that once YOYO-1 intercalates into dsDNA, it emits florescence that can be measured by a confocal microscope (Supplementary Table S1). If the chemical conditions tested can induce separation of stretches of dsDNA leading to YOYO-1 release, one would expect the fluorescence signal intensity to (i) progressively diminish during DNA denaturation, and (ii) remain low with no complete recovery upon reintroduction of the original chemical conditions (a washing step). Importantly, a change of chemical conditions may also modify photophysical properties of the dye, and influence brightness (i.e. the product of quantum efficiency and absorption cross section). In such a case, however, we would expect the signal to respond immediately and then remain stable over-time.

Testing the aforementioned chemical factors we found that an addition of DMSO resulted in an immediate increase and a subsequent period of stable intensity of the YOYO-1 signal, whereas after exposure to BME the signal did not change (Figure 1A). In contrast, addition of MEA results in an immediate signal decrease followed by an approximately constant signal level. These changes in fluorescence signal levels are presently not understood, considering that BME and MEA are similar in structure and function. Both are primary thiols used for induction of a ‘dark-state’ in SMLM (21,37,38). Washing or reperfusion has previously been reported to facilitate recovery of fluorescence emission of several dyes from a light-induced non-emitting state formed in the presence of a thiol (39). In our experiments, a few washing steps following incubation with MEA resulted in signal restoration to almost 100% (for 333 mM MEA) or at least 85% of the initial value (for 666 mM MEA).

Subsequently, we evaluated the effect of acidic pH on nuclear YOYO-1 fluorescence signal. While pH of 4.32 had no effect on the YOYO-1 signal over time (except for an initial increase in signal intensity), pH 3.70 induced a gradual fluorescence signal decrease within minutes after addition of ascorbic (AA, Figure 1B) or hydrochloric acid (HCl, Figure 1C). This indicates the primary role of low pH in decreasing the intensity of YOYO-1 signal. The role of ascorbic acid as a reducing agent (23) is thus expected to be secondary, because the decrease in a YOYO-1 signal is similar when adding hydrochloric or ascorbic acid, although they differ in redox potentials.

Small stretches of a DNA double helix are known to undergo constant, reversible transitions between single- and double-stranded structure (DNA breathing, (40)) due to a dynamic nature of hydrogen bonds between complementary bases. These conformational changes are influenced by changes in the concentration of hydrogen ions. Nitrogenous bases are hydrogen donors necessary to form these hydrogen bonds. The p*K*_a_ value of their amine groups are 4.2 and 3.4 (41). Acidification of the environment leads to protonation of the bases, and changes in their electron structure. At moderately low pH, DNA undergoes conformational changes due to base protonation preceding denaturation (42). Studies on DNA in acidic conditions indicated DNA structure instabilities (43) and an increasingly disordered DNA structure with decreasing pH (44). Strongly acidic environment eventually leads to breakage of hydrogen bonds and an irreversible loss of double-stranded structure.

It has been previously shown that homodimeric cyanine DNA-binding dyes have negligible photon emission in the presence of denatured (single-stranded) DNA (33) and that they associate with DNA immediately upon ssDNA hybridization (45). Taking this into account, the decrease of YOYO-1 fluorescence intensity apparent in Figure 1B and C might be the result of a release of the dye from the complex with the DNA (for structure of the complex see PDB: 108D) or a change in molecular structure of the molecule of DNA-bound YOYO-1. Worth noticing is also the fact that at the end of the experiment we recovered ∼20–30% of the YOYO-1 signal through a restitution of the original conditions (wash). Nevertheless, 30% (ascorbic acid, Figure 1B) and 60% (hydrochloric acid, Figure 1C) of the YOYO-1 signal was not recovered. This prompted us to conclude that the unbound YOYO-1 was washed away, but some YOYO-1 stayed bound to the remaining dsDNA. This is in line with our recent finding that even exposing DNA in fixed cells to pH <3, denaturation is not complete (46). Lastly, similar results have been obtained for other cyanine dyes in the presence of low pH, primary thiols (Supplementary Figure S2), and also in buffers of an increased ionic strength (Supplementary Figure S3, discussed in Supplementary Note 1).

To summarize, we identified a low pH, MEA (Figure 1A, B, C, Supplementary Figure S2), and an increased ionic strength (Supplementary Figure S3) as potentially useful for strong reduction of the fluorescence signal obtained from DNA-binding dyes. Next, we tested whether this may be used to achieve optical isolation of single fluorescent molecules, which is necessary for SMLM.

### DNA structure fluctuation-assisted BALM—experimental design

We tested MEA for its ability to induce optical isolation of DNA-binding dyes in cell nuclei, but found that the SMLM images obtained were of poor quality (Supplementary Figure S5). In Figure 1 and Supplementary Figure S2, we demonstrate that acidic buffers are able to decrease the nuclear fluorescence intensity for all three dyes tested more effectively than MEA (approx. only 20% signal remained in comparison to 45% for MEA). Considering that DNA chemistry in environments of different acidity has been well characterised, we chose to evaluate the usefulness of acidic conditions for DNA structure fluctuation-assisted BALM (fBALM).

**Figure 2.**
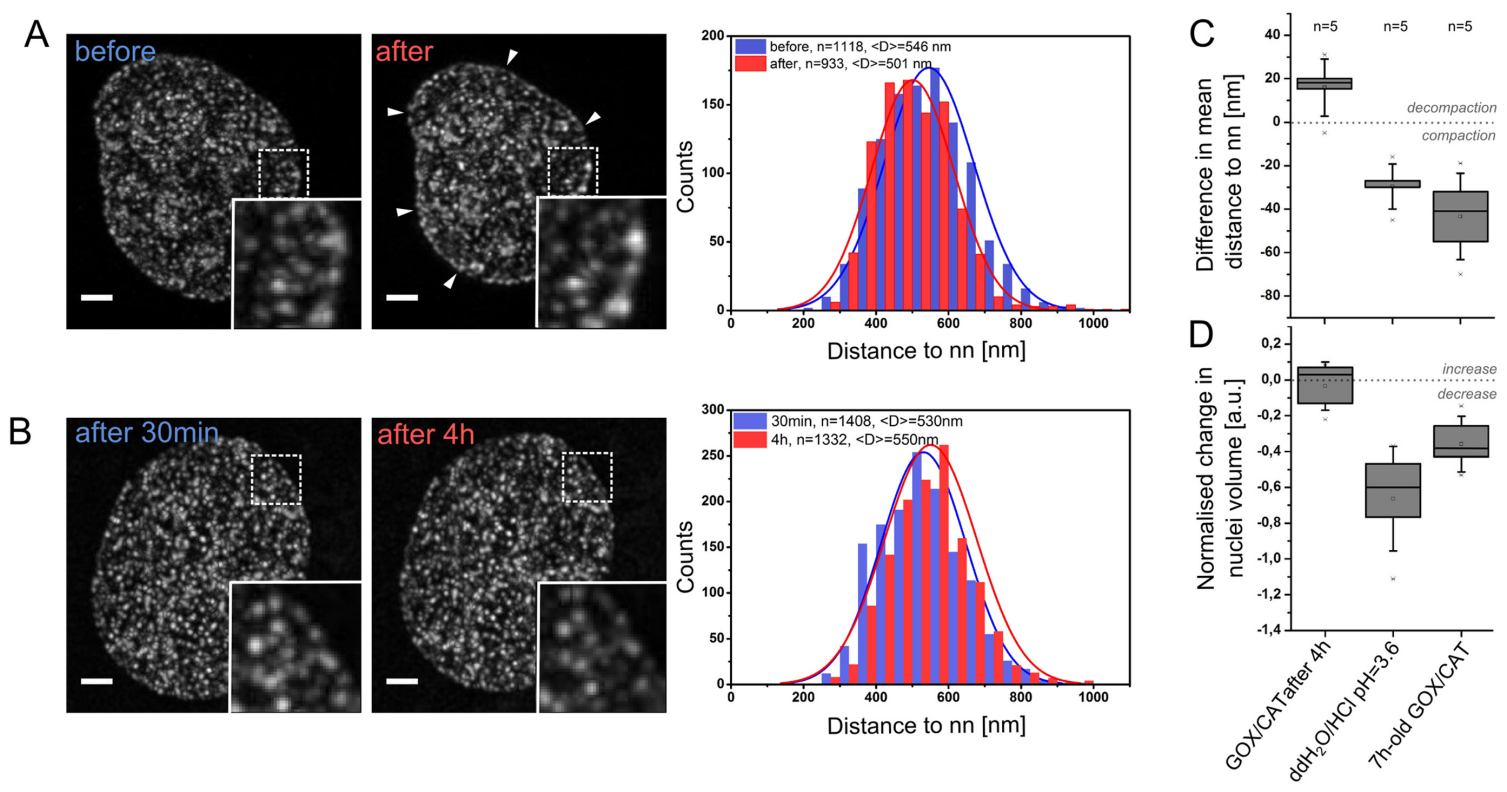
Effect of acidic imaging media on chromatin structure and nuclear volume. Nascent DNA was labeled with Alexa 555, and imaged by 3D confocal microscopy followed by image deconvolution. (**A**) Nearest neighbor (nn) analysis of Alexa 555 foci before and after 10 min of incubation in ddH_2_O/HCl buffer of pH 3.6, demonstrating shrinking of cell nuclei (arrows). (**B**) Cells were embedded in localization-microscopy buffer containing glucose oxidase, catalase and 10% glucose. 3D confocal microscopy was performed immediately after embedding (pH close to neutral) and after 4 h (pH ∼ 3.7, for details see Figure 3A, Supplementary Figure S4). Maximum Z-projections are presented. Scale bar, 2 μm. Centroid fitting enabled sub-pixel accurate foci localization and nearest neighbor (nn) analysis of chromatin-bound reference ‘points’. Representative examples of graphs are presented. The number of foci before and after the treatment with the respective average distance <*D*> are indicated. (**C**) Difference in an average nearest neighbor distance for: (i) an addition of ddH_2_O/HCl (pH 3.6, as in A); (ii) imaging buffer containing glucose oxidase undergoing acidification over 4 h (final pH ∼ 3.7, as described in B); and (iii) an addition of a 7 h-old imaging buffer containing glucose oxidase (pH ∼ 3.7). (**D**) Normalized change in nuclear volume for respective conditions. Crosses correspond to outliers, squares denote mean value, boxes indicate the 25 and 75 percentile range. Nn analysis of >15 000 Alexa 555 foci in total was performed in 15 cells.

A precondition to successful single molecule localization imaging with resolution down to a few tens of nanometres is preservation of the nanostructures of interest. Based on previous experience with 3D-fluorescence *in situ* hybridization (47), we speculated that an addition of an acidic buffer (pH < 4) to fixed cells may have a profound and undesired influence on the structure of the cell nucleus. In order to verify this notion, we designed a quantitative assay to investigate structural changes potentially exerted by low pH imaging buffer. This procedure was based on selective labeling of DNA with EdU in replicating cells and resulted in small, diffraction limited, fluorescence reference spots scattered throughout the nucleus. The 3D positions of these foci were extracted from confocal microscopy images. Taking advantage of the same localization principles valid for SMLM, we determined their positions in 3D with nanometer precision using a center of gravity approach, and calculated the histograms of the nearest neighbor (nn) distances before and after the acid treatment (Figure 2A). This quantitative analysis revealed shortening of average nearest neighbor distances by ∼50 nm, resulting from shrinking of the cell nuclei by ∼40% following addition of an acidic buffer (Figure 2D) (the buffer indicated as potentially useful for BALM, Figure 1C). Such relatively large changes can defeat the purpose of super-resolution imaging of the cell nucleus. Moreover, straight addition of acidic buffers to the sample yielded low density of the extracted single molecule signals due to a high background signal, a sparse distribution of fluorescent bursts, and a rapid photobleaching (Supplementary Figure S5). Altogether, these aspects eliminated this procedure from further investigations.

We reasoned that in order to reduce the aforementioned structural artifacts the change of the pH should be introduced gradually over time. In order to address this issue we decided to employ glucose oxidase (GOX) and catalase (CAT) enzymatic oxygen scavenging system. GOX incorporates molecular oxygen into a complex with glucose, hence decreasing the solution's oxygenation within minutes (48) and preventing fluorophore photobleaching. As an interesting side-effect, a d-glucono-1,5-lactone is formed and rapidly hydrolysed to gluconic acid (p*K*_a_ = 3.7) that in turn contributes to the gradual acidification of the buffer (49).

**Figure 3.**
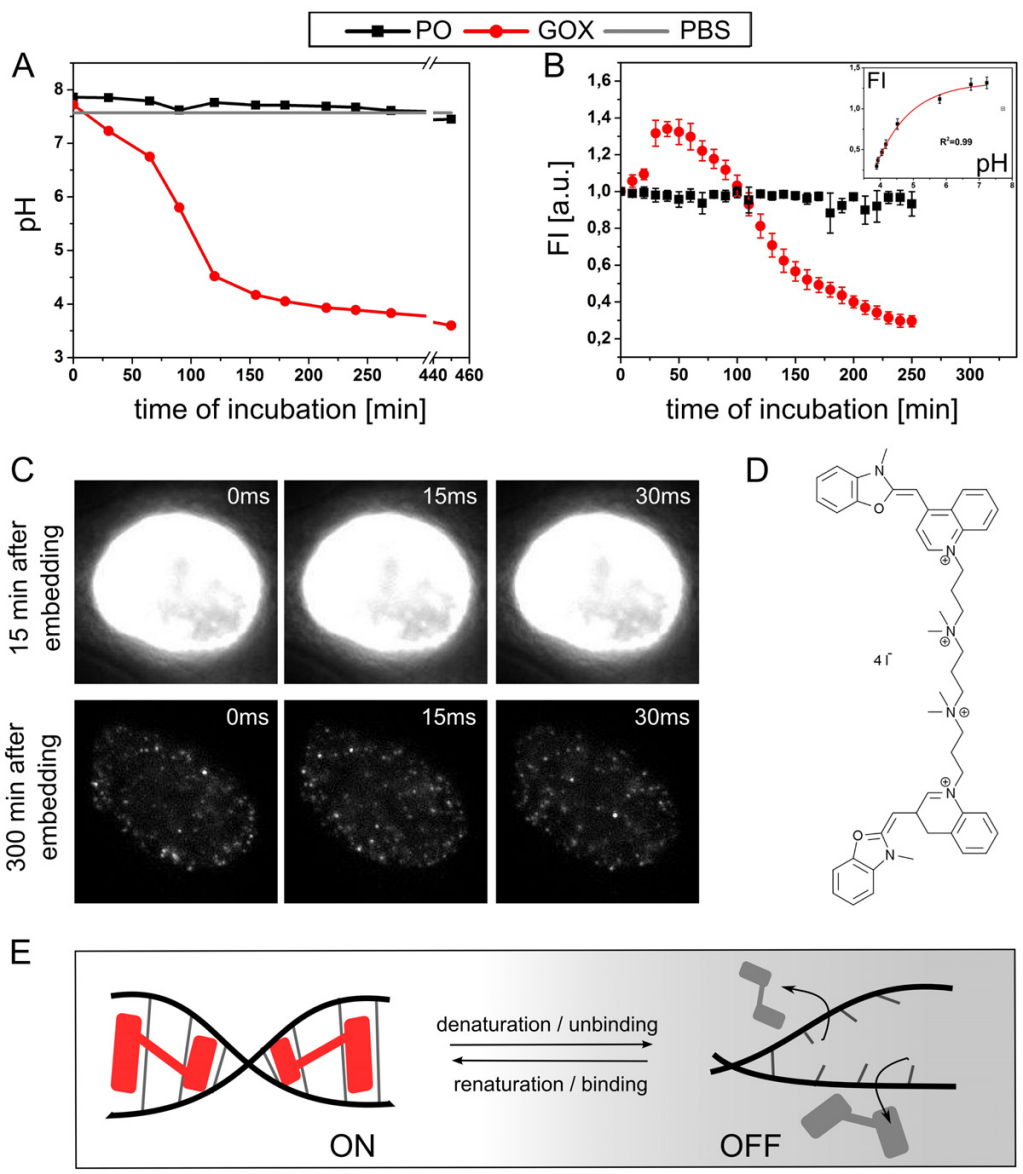
DNA structure fluctuation-assisted BALM using YOYO-1 in acidic conditions. (**A**) Time course pH measurements of the SMLM buffers containing oxygen scavenging enzymes: glucose oxidase (GOX) or pyranose oxidase (PO). (**B**) Time course confocal microscopy of cells (cellular fluorescence signal, arbitrary units) stained using 10 nM YOYO-1 embedded either in a buffer containing GOX or PO. The inset shows the fluorescence intensity vs. pH for the GOX-containing buffer. The initial point (cross) has not been utilized for exponential fitting. See relevant Supplementary Figure S4. (**C**) Raw single molecule localization microscopy acquisition frames 15 min after embedding the sample in glucose oxidase and catalase containing buffer (upper row) and after roughly 5 h (lower row). Frames were taken every 15 ms; however, the read-out time of the CCD camera is not taken into account here. Cells were exposed to ∼1 kW/cm^2^ focused 491 nm laser excitation. No similar behavior was detected for PO. (**D**) Molecular structure of YOYO-1 showing two aromatic groups responsible for DNA double helix bis-intercalation. (**E**) A scheme depicting the proposed mechanism underlying fBALM methodology. Local DNA structure instability at pH∼3.7 induces a change in dye-DNA interaction (potentially dissociation), resulting in a strongly reduced fluorescence (gray shaded molecules). While the DNA undergoes transient renaturation to the double stranded form, the dye molecules in the vicinity immediately associate with the polymer and become highly fluorescent (45) (indicated in red) until bleached or dissociated again (gray shaded). Under appropriate conditions, dye molecules decorate DNA only sparsely and emit detectable fluorescence (see C, bottom row). For the detailed DNA–YOYO-1 complex structure see PDB: 108D.

On the basis of these considerations, we studied the effect of these enzymes on pH and YOYO-1 signal intensity in cells over time (Figure 3A, B, Supplementary Figure S5). Our experiments revealed that after ∼180 min of incubation of the enzymes and glucose in such a buffer, the pH drops below 4 and after 300 min remains constant at around ∼3.7 (Figure 3A), i.e. a value for which DNA structural changes occur (42,43,50). The fluorescence signal of YOYO-1 labeled cells in sealed samples measured using time-lapse confocal microscopy demonstrates an initial increase followed by a decrease to about 20% of the initial value (Figure 3B) along with the drop in pH (Figure 3A).

This time, our quantitative analysis of the nuclear structure alteration upon gradual decrease of pH showed a substantially reduced effect on the nuclear structure as measured in nearest neighbor distance analysis and in the nuclear volume (Figure 2B–D). Note, that the positive change in the mean nearest neighbor distance is a result of a slight bias since the number of signal maxima identified after incubation with all the buffers tested was always lower by ∼10%. This means that this analysis will be skewed towards an increase in the mean nearest neighbor (nn) distance value. Nevertheless, in the case of an abrupt addition of an acidic buffer the change was always negative and significantly larger than when the GOX/CAT system used.

Next, we studied the behaviour of YOYO-1 in cell nuclei submerged in GOX/CAT buffer by exposing them to a high intensity 491 nm laser illumination (∼1 kW/cm^2^) using a widefield microscope in single molecule localization mode. Exposure to light soon after the cells were embedded in a freshly prepared imaging buffer and sealed resulted in fluorescence signal saturation. However, after an additional ∼300 min, the YOYO-1 fluorescence behaviour dramatically changed: immediately after the light exposure, isolated, strong diffraction-limited signals appeared indicating the potential of this preparation method for SMLM (Figure 3C). At pH 4, GOX activity is still at ∼40% of its value at neutral pH (51), therefore GOX will keep the oxygen concentration low in a sealed sample in acidic pH. When GOX was replaced with pyranose oxidase (PO), whose product of oxygen scavenging does not have an effect on pH of the buffer (49) (Figure 3A), the YOYO-1 signal intensity remained constant over time (Figure 3B) and no single molecule signals could be observed (data not shown).

### DNA structure fluctuation-assisted BALM of HL-1 cells

**Figure 4.**
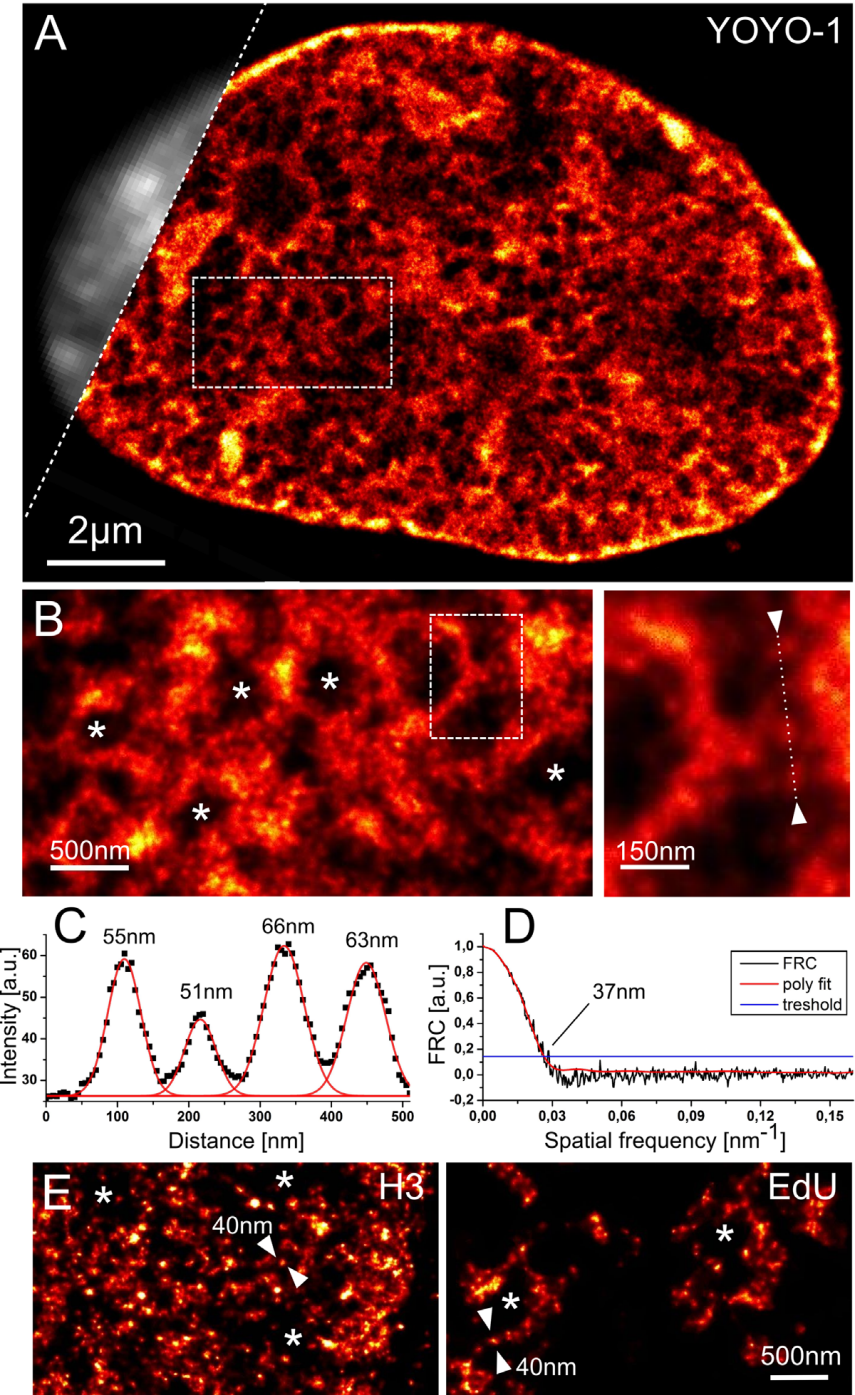
DNA structure fluctuation-assisted BALM (fBALM) of HL-1 cell nucleus using YOYO-1. (**A**) Super-resolution DNA signal density image reconstructed from single molecule signals of YOYO-1 transiently fluorescing at locally renaturing dsDNA. The image was acquired 5 h after immersion with glucose oxidase containing buffer (pH∼3.7). The gradual drop in pH over time ensured preservation of nuclear structure (Figure 2). A part of a conventional wide field image presented in grey for comparison. (**B**) Left: 3× magnification of a rectangular region of interest indicated in (A). Asterisks show void regions with very low signal density, likely an interchromatin compartment (3). Right: high magnification of the small inset shown in (B) on the left depicts a progressively enlarged part of the image illustrating structure details at nanometer scale. (**C**) Signal intensity profile between arrow-heads marked in B, inset lower right. The red line corresponds to multiple Gaussian fits. Black squares correspond to the actual signal intensity in the DNA density image. The numbers correspond to the full-width-at-half-maximum (FWHM) for each of the peaks fitted with Gaussian curve. (**D**) Fourier ring correlation (FRC) analysis of the super-resolution image with the resolution value estimate obtained from the intersection of the polynomial fit with the empirical threshold. (**E**) Comparison of single molecule localization microscopy on HL-1 cells labeled with anti-H3 primary and Alexa 647 secondary antibody (left), or with a 10 min EdU pulse followed by Alexa 488 ‘click-it’ reaction to stain chromatin surrounding replication factories.

**Figure 5.**
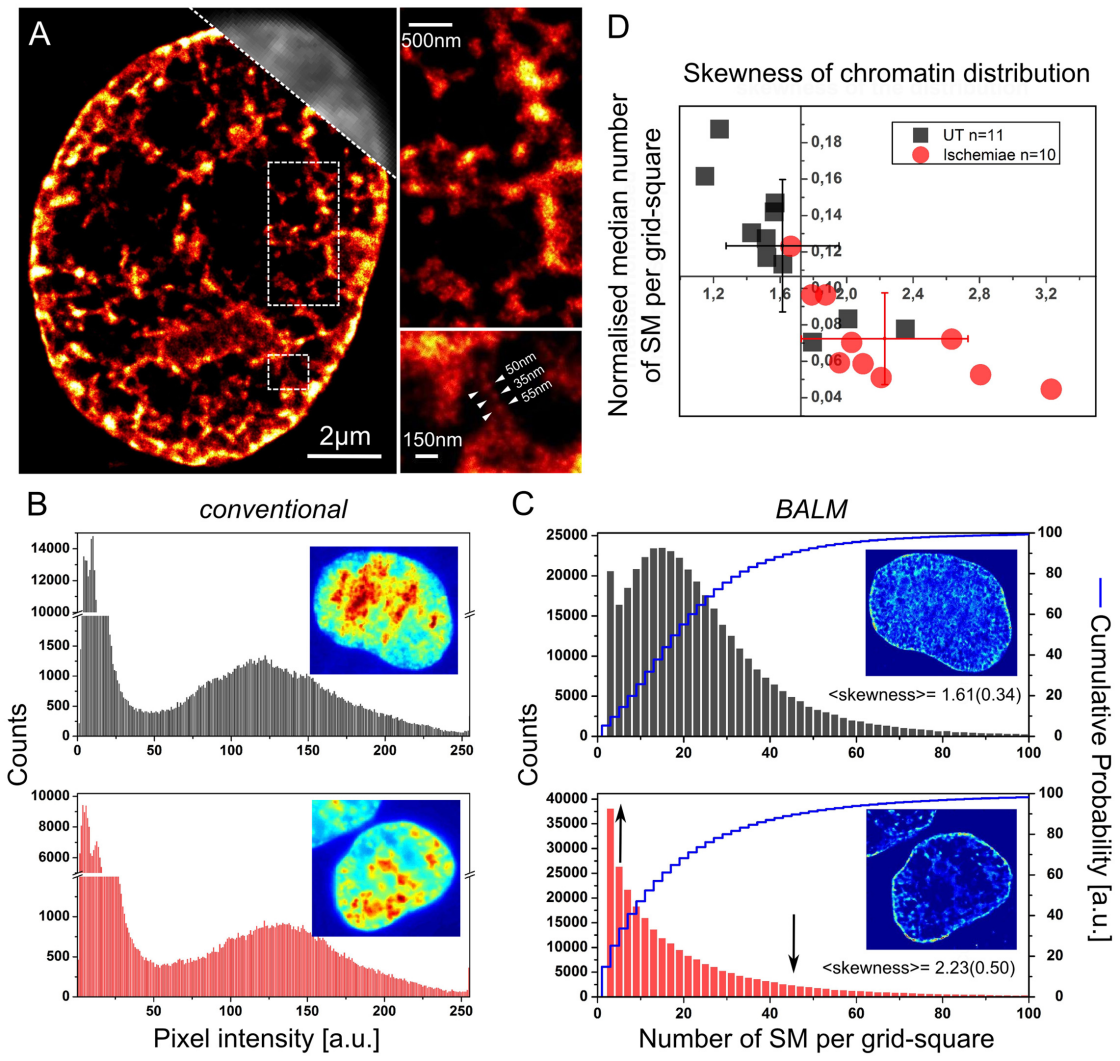
DNA structure fluctuation-assisted BALM (fBALM) reveals a change of chromatin density distribution upon transient ischemic treatment. (**A**) Super-resolution DNA signal density image reconstructed from single molecule signals of YOYO-1 originating from the DNA of a cell subjected to a model ischemia. Conventional resolution widefield image in grey (upper, right corner) shown for comparison. Dashed lines indicate rectangular regions zoomed in and presented on the right. Arrowheads highlight a fiber-like structure. For more details see Supplementary Figure S11. (**B**) YOYO-1 signal intensity distribution in conventional images of untreated (upper panel, black) and ischemic cells (lower panel, red). Peak at low values <50 originates from the low-intensity background surrounding the nucleus. (**C**) Single molecule YOYO-1 signal distribution in untreated (upper panel, black) and ischemic (lower panel, red) nuclei obtained using fBALM. Respective cumulative probability presented in blue (step-curves). Single molecule positions were binned in 2D space into 60 × 60 nm^2^ grids. Eleven cells for untreated and 10 cells for ischemia were analysed. Black arrows indicate the tendency of the alteration to the chromatin density distribution caused by ischemia (either increase or decrease). Insets correspond to examples of cell images; color-coding reflects the signal intensity in conventional images and localization density in fBALM images. (**D**) fBALM analysis; normalized median number of single molecule signals (SM) per grid-square plotted against respective chromatin density distribution skewness (a measure of the asymmetry of the data around the sample mean). Each symbol represents values measured for single cells. Error bars indicate standard deviation around mean value for untreated (UT) and ischemic cells.

Having established the basis for DNA structure fluctuation-assisted BALM (fBALM), we performed experiments aiming at an investigation of the nuclear chromatin organisation in its native state at enhanced resolution. We employed a well characterized HL-1 murine cardiac muscle cell line (26). Cells were stained using YOYO-1, washed and submerged in a buffer containing glucose oxidase. Single molecule localization acquisition was started after 3 h resulting in a detection of ∼1.0 × 10^6^ single molecule signals (SM) in an individual cell nucleus, using a total of 30,000 frames (acquisition time of ∼35 min, Figure 4A). Assuming that a single imaging plane through a cell nucleus comprises one tenth of the nuclear volume, this gives 10^6^ observations per 10^8^ DNA basepairs (bp), i.e. an average coverage of 1 per 100 bp. In comparison YOYO-1 is capable of binding to dsDNA every 3.2 ± 0.6 bp (32). Using Fourier ring correlation (FRC) analysis (29,30) we estimated the overall structural resolution in this image to reach ∼37 nm (Figure 4D) whereas the smallest structures found ranged from 35 to 50 nm at full width at half maximum (Figures 4C and 5A, for a more detailed discussion see Supplementary Note 2).

It has not escaped our attention that the chromatin in the nucleus seems to be arranged in a structure resembling a ‘spider's web’. Dense signal accumulations at intersections in the chromatin network typically have a diameter between 100 and 400 nm and separate void (DNA sparse) regions of similar size (Figure 4B, asterisks). Interestingly, these structures observed in murine cardiac muscle cells apparently are composed of small granular units (Figure 4C) that qualitatively bear a strong resemblance to H2A, H2B and H3 tagged nucleosome clutches revealed lately in primary human fibroblasts using STORM (15). For comparison, we produced such SMLM data for HL-1 cells with immunofluorescently labeled histone H3 with a secondary antibody conjugated with Alexa 647, and with the DNA base analogue 5-ethynyl-2΄-deoxyuridine (EdU) conjugated with Alexa 488 (52,53). (Figure 4E, for more details on comparison of these chromatin labelling strategies and potential limitations see Supplementary Table S2).

In addition, we set out to utilise the fBALM protocol to image the nuclear periphery. It is a highly structured arrangement closely interacting with lamin meshes through lamin associated domains (reviewed in (9)). fBALM proved to be successful in capturing the sub-diffraction information about locations of the entrances to the interchromatin compartment (largely DNA free space, for review see (3)) and thus about the anticipated location of nuclear pore complexes (Supplementary Figure S7). In this case we took advantage of the limited YOYO-1 penetration towards the centre of the cell nucleus, in order to image only the single dye molecules binding at the nuclear periphery with a high signal to noise ratio, i.e. reduction of the signal from an interior of the cell nucleus.

### Effect of experimental ischemia on nuclear structure

Ischemia, or oxygen and nutrient deprivation, is known to reduce ATP levels in cells and, as a result, leads to transcription downregulation in the great majority of genes (16). In addition, there is evidence that repressed and inactive genes are more compacted, i.e. they occupy a smaller space than the active ones (9,17). Thus, we proceeded with an ischemic model of HL-1 cells and employed DNA structure fluctuation-assisted BALM to study nuclear structures in the vastly inactivated genome (16).

First the HL-1 cells were subjected to hypoxia combined with hypoglycemia for 1 h (see Materials and Methods), fixed and stained with YOYO-1. Next, they were immersed in an imaging buffer with GOX- and CAT-containing a low pH oxygen scavenging system for at least 3 h, as described above (Figure 3).

The fBALM images of DNA in ischemic cells revealed massive retraction of chromatin to the nuclear periphery as well as formation of dense chromatin clusters with a diameter of ∼300 nm, scattered throughout the nucleus with a concomitant expansion of a DNA-free space (Figure 5A). Perichromatin, the chromatin surface contacting the interchromatin compartment believed to be a region harboring DNA transcription and replication (3,54), underwent a significant reduction as chromatin condensed. Indeed, the transcription rate under these conditions was reduced by an order of magnitude (16).

In order to address quantitatively the subject of an overall chromatin arrangement in ischemic nuclei we calculated the pixel intensity frequency for the 2D binned histogram reconstructions (see extended Materials and Methods). Figure 5C shows the results obtained with fBALM in untreated and ischemic cells. The analysis indicates an evident change in the density distribution revision upon ischemia (black arrows). The lower median number of SM per grid-square (Figure 5D, ordinate) might stem from a higher occupancy of the potential dye binding sites with native polycationic compounds and divalent cations, as hypothesized before (16). Lastly, this evaluation method enables a direct comparison of fBALM data with conventional widefield images, as in the latter the intensity of a pixel can be safely assumed to reflect directly the number of fluorescing YOYO-1 molecules bound to the DNA and hence can be similarly binned in a histogram of intensities. In this case, however, we failed to observe different DNA-density distribution in ischemia (Figure 5B), pointing to the fact that the diffraction limited imaging method, unlike SMLM based fBALM, is insufficient to observe subtle hypoxia-induced changes in chromatin structure.

### fBALM resolves different chromatin distribution in different cancerous cells

Next, we set out to conduct fBALM imaging of a particular nature of the DNA structure in Hodgkin's lymphoma (H) cells. The malignant mono- or bi- and multinucleated Reed–Sternberg (RS) cells in HL patients’ blood are considered a hallmark of this disease and are used for a microscopy-based diagnosis. Not only a multitude of cellular functions are affected in RS cells (55) but also the amount of translocations increases with the number of subnuclei (56). fBALM images of Hodgkin's lymphoma bi-nucleated RS cells revealed that their structure varied immensely from the structure of cardiomyocytes (Figure 4). The H nuclei are smaller, contain more DNA-free spaces, and their boundaries have a very complex shape, including boundary invaginations (Supplementary Figure S8, arrows).

Interestingly, super-resolution (fBALM) images of neuroblastoma, a human infant cancer arising from neural crest, reveal again a distinct texture. While the nuclear boundary has a very similar appearance as in the case of cardiomyocytes, i.e. no invaginations are present, the internal nuclear structure is rather sparse. Interestingly, differences in coarse structures become apparent in fBALM images (compare with widefield acquisitions, Supplementary Figure S8). We attribute this fact to an improved sectioning capability of fBALM compared with a confocal microscope (Supplementary Figure S6).

## DISCUSSION

The DNA structure fluctuation-assisted BALM (fBALM) approach described in this report made it possible to create single molecule localization images of the distribution of YOYO-1 interacting with nuclear DNA in mammalian cells with a structural resolution in the tens of nanometre range.

To further boost the resolution in fBALM one could consider a modification to the excitation scheme using a selective plane illumination perpendicularly oriented to the detection direction (57). The advantage of using this add-on along with the fBALM scheme would be 2-fold: (i) light sheet illumination of the cell nucleus enables higher localization precision with the same photon number due to the suppressed out-of-focus fluorescence which typically brings about an increase of the image background (58), and (ii) light-sheet combined with fBALM would potentially reduce the bleaching-induced losses in the intranuclear dye pool (as only a single plane instead of the whole nucleus would be subjected to excitation and photobleaching) enabling prolonged acquisitions resulting in higher achievable localization density and likely a better uniformity of the ‘sampling’ of the illuminated plane. These factors are expected to increase fBALM reconstruction quality and the achievable resolution, and could be useful for 3D imaging. Nevertheless, in this study we demonstrate the significant utility of fBALM using the basic epi-fluorescence microscopy setup only.

An example of fBALM application presented here includes imaging of chromatin structure induced by changes of the environment, as demonstrated in an ischemic treatment. Manifold nuclear factors (17) as well as physical principles (59) are already known to have an impact on chromatin compaction and nuclear structure. This knowledge, however, could be broadened by utilising fBALM for investigations of the interplay between chromatin structure, epigenetic factors, and gene regulation on the nanoscale, and in a whole cell nucleus.

Our fBALM DNA imaging data, combined with available genome wide chromatin data, prompts us to conclude that a classical chromatin model based on the existence of euchromatin and heterochromatin, assuming a simple variation in local chromatin concentration by a factor of 3–4 (60,61) might be misguiding. In contrast, fBALM data aided by a high specificity of the dye used to image the DNA (Supplementary Figure S10) and careful handling of the background signals (see extended Materials and Methods in supplementary information) indicates essentially no DNA-originating signal in the interchromatin compartment (Figures 4 and 5, Supplementary Figure S4). This observation is in agreement with the results of the previous microtome serial block face scanning electron microscopy studies (62). We note that a typical conventional fluorescence microscopy of nuclear DNA detects signal variations of ∼3 fold only (see Figures 4, 5 and Supplementary Figure S6C).

We anticipate that fBALM may contribute to unravel new facts pertinent to chromatin structure at various levels of organisation, including the internal nanostructure of the hypothesized topologically associated domains (TADs) *in situ*, formation of the so called ‘meta-TAD’ arrays (6), and finally to test a model of active-inactive chromatin compartment organisation (3). Importantly, methods to label specific genomic loci already exist and, in combination with fBALM, they may provide complementary approaches to studies of specific gene-related structural changes.

It is worth emphasizing that the native chromatin structure reflects cell fate and function (e.g. see (14)) or cancer disease progression (63). On a single cell level, currently fBALM constitutes one of a few microscopy-related methodological candidates (along with structured illumination microscopy, SIM) that can significantly contribute to this field. It is expected that a wealth of knowledge that may be accessible with fBALM contributes to the further development and application of fluorescence microscopy of DNA-binding dyes, including detailed studies and better mechanistic understanding of dye binding to DNA and dye photophysics, such as fluorescence quantum yield enhancement, sequence specificity in DNA instability (33), dye photo-blinking, and chemical modifications of the DNA-binding dye structure (for more details see Supp. Note. 4). Nevertheless, this method is very competitive in providing a high nanostructural resolution of the nuclear DNA distribution as compared to e.g. anti-histone antibody staining where antibody accessibility is limited thereby preventing labeling of dense chromatin sites (16,46,64), see Supplementary Table S2 for comparison of various methods of chromatin labeling for SMLM).

We cannot fully rule out that the conditions that were indicated as useful for DNA structure fluctuation-assisted BALM, also influence to some extent the chemical properties of the DNA-binding dyes, and as a consequence modify the interaction with the single stranded and double stranded DNA. Likely, the effect of both, DNA structural changes and, to some extent, additional protonation/reduction of the DNA-binding dye, influence their reciprocal interactions, enabling successful DNA super-resolution structure fluctuation-assisted BALM imaging.

The conditions studied in this report are of general importance in single molecule methods employing transient binding (24), in particular using transient DNA hybridization just as DNA-PAINT (65) or motion blurred PAINT (45) where typically the length of hybridizing ssDNA probes, sequence mismatch, or ionic strength of a buffer are carefully adjusted to ensure transient interaction. Manipulating pH of the environment would constitute an additional exciting aspect of these approaches.

## CONCLUSIONS

In this study, we found that YOYO-1, a dsDNA bis-intercalator, diffuses throughout the cell nucleus relatively slowly and, upon gradual introduction of denaturing conditions, using a pH near the value of 3.7, undergoes a fluorescence signal reduction enabling optical isolation and single molecule ‘sampling’ of DNA. This novel procedure, termed ‘DNA structure fluctuation-assisted BALM’ (fBALM) permitted us to image DNA in the cell nucleus with an average structural resolution in the order of 50 nm. To our knowledge, this methodology presently constitutes the most accessible super-resolution DNA microscopy based on single molecule signal detection in the cell nucleus.

## Supplementary Material

Supplementary Data
