# Supplementary material for: Imaging chromatin nanostructure with binding-activated localization microscopy based on DNA structure fluctuations
Source: Nucleic Acids Res. 2017 Jan 13;45(8):e56. doi: 10.1093/nar/gkw1301 (PMC5416826; doi:10.1093/nar/gkw1301)
Supplement: Supplementary Data [file gkw1301_supp.docx]

**Imaging chromatin nanostructure with binding activated localisation microscopy based on DNA structure fluctuations**

**Supplementary Materials**

**Aleksander Szczurek^1^, Ludger Klewes^2^, Jun Xing^1^, Amine Gourram^1,3^, Udo Birk^1,3^, Hans Knecht^4,5^, Jurek W. Dobrucki^6^, Sabine Mai^2^, Christoph Cremer^1,3,7*^**

^1^Institute of Molecular Biology, 55128 Mainz, Germany;

^2^University of Manitoba, Cancer Care Manitoba, Winnipeg, R3E 0V9, Canada;

^3^Physics Department University Mainz (JGU), 55128 Mainz, Germany;

^4^Département de Médecine, CHUS, Université de Sherbrooke, 3001-12e Avenue Nord, J1H 5N4 Sherbrooke, Québec,Canada ;

^5^Department of Medicine, Jewish General Hospital, McGill University, 3755 Côte-Ste-Catherine Road, H3T 1E2 Montreal, Québec ;

^6^ Department of Cell Biophysics, Faculty of Biochemistry, Biophysics and Biotechnology, Jagiellonian University, Kraków, Poland;

^7^Kirchhoff Institute of Physics (KIP), and Institute of Pharmacy & Molecular Biotechnology (IPMB) University Heidelberg, Germany;

*corresponding author Christoph Cremer, +49 6131 3921518, [c.cremer@imb-mainz.de](mailto:c.cremer@imb-mainz.de)

__________________________________________________________________________________


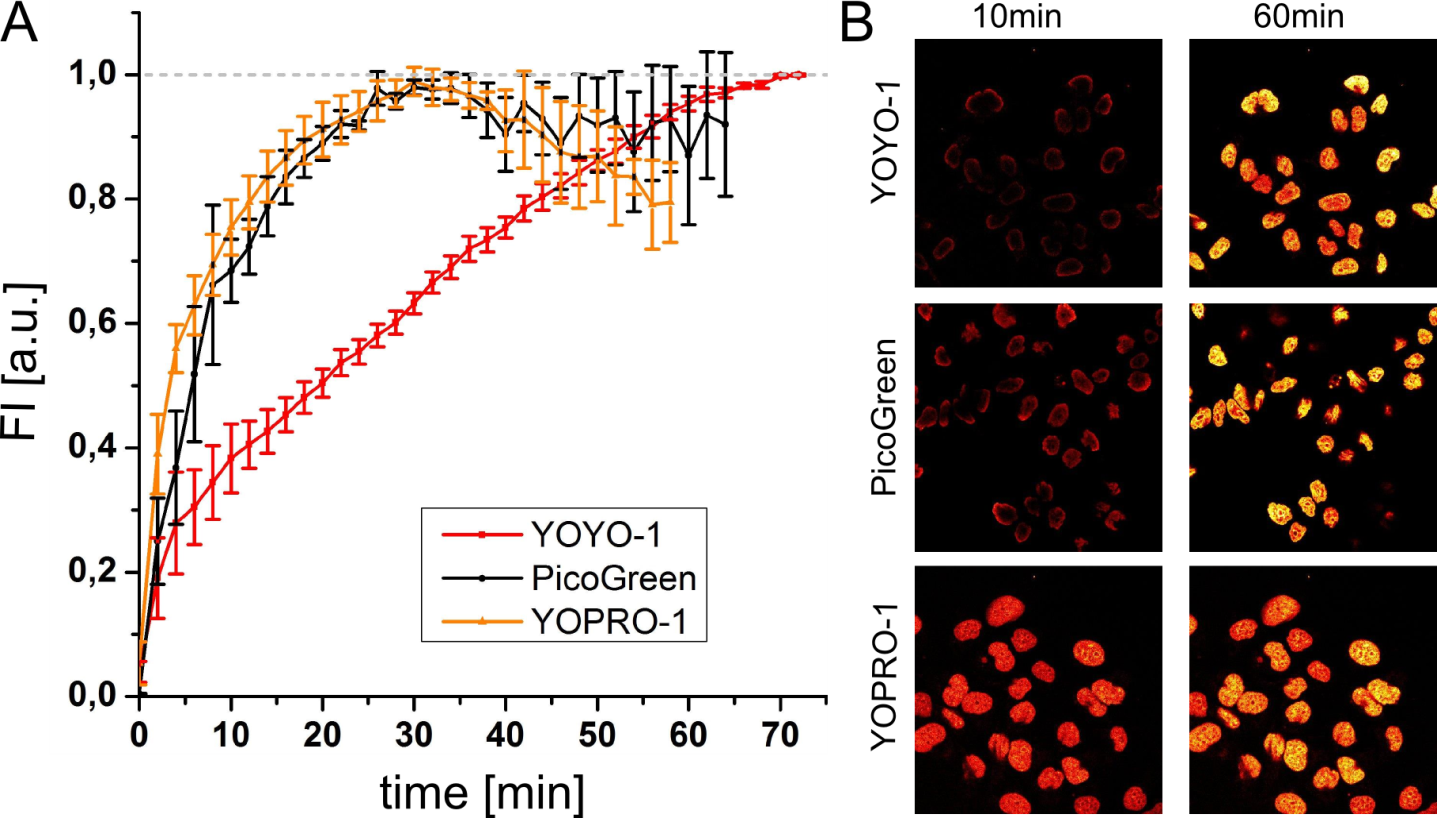


**Supplementary Figure S1. Kinetics of association of YOYO-1, PicoGreen and YOPRO-1 with cellular DNA in fixed cells.** RNase-treated HeLa cells were exposed to 12 nM YOYO-1, 1:10,000 PicoGreen or 30 nM YOPRO-1. Changes of the mean nuclear fluorescence intensity (FI; arbitrary units) with time were measured using a confocal microscope. **A**) Fluorescence intensity (FI) from 5 to 6 fields-of-view plotted as a fraction of the saturation intensity (normalised to 1.0 for a given cell nucleus) versus the time elapsed since the beginning of incubation with the dye; error bars correspond to the standard deviation. Among the three dyes studied only for YOYO-1 the fluorescence signal does not reach a plateau and a stationary state within 1h of the experiment, whereas the FI for both YOPRO-1 and PicoGreen reached the maximum intensity already after ~30 min from the beginning of incubation. The slight FI decrease after ~35 min for YOPRO-1 and PicoGreen might be ascribed to photobleaching during the experiment or to self-quenching due to a high dye concentration (we observed the latter, when high concentrations were used, *data not shown*). Deviations from an expected exponential growth-like tendency are likely to arise from the experimental design i.e. due to the imaging at the central section of flattened ellipsoidal nuclei and due to hindered dye accessibility to the interior of the nucleus. **B**) Examples of images taken 10 and 60 min after adding the dye. Note that the limited capacity to penetrate to the nucleus interior, in particular in the case of YOYO-1, is manifested in the initial preferential staining of the nuclear periphery and a lack of signal from the innermost regions. This demonstrates one of the limits of externally supplying the DNA binding dyes in BALM imaging of the entire cell nucleus (i.e. supply of a pool of dye in the embedding media), especially when using insufficient incubation times.


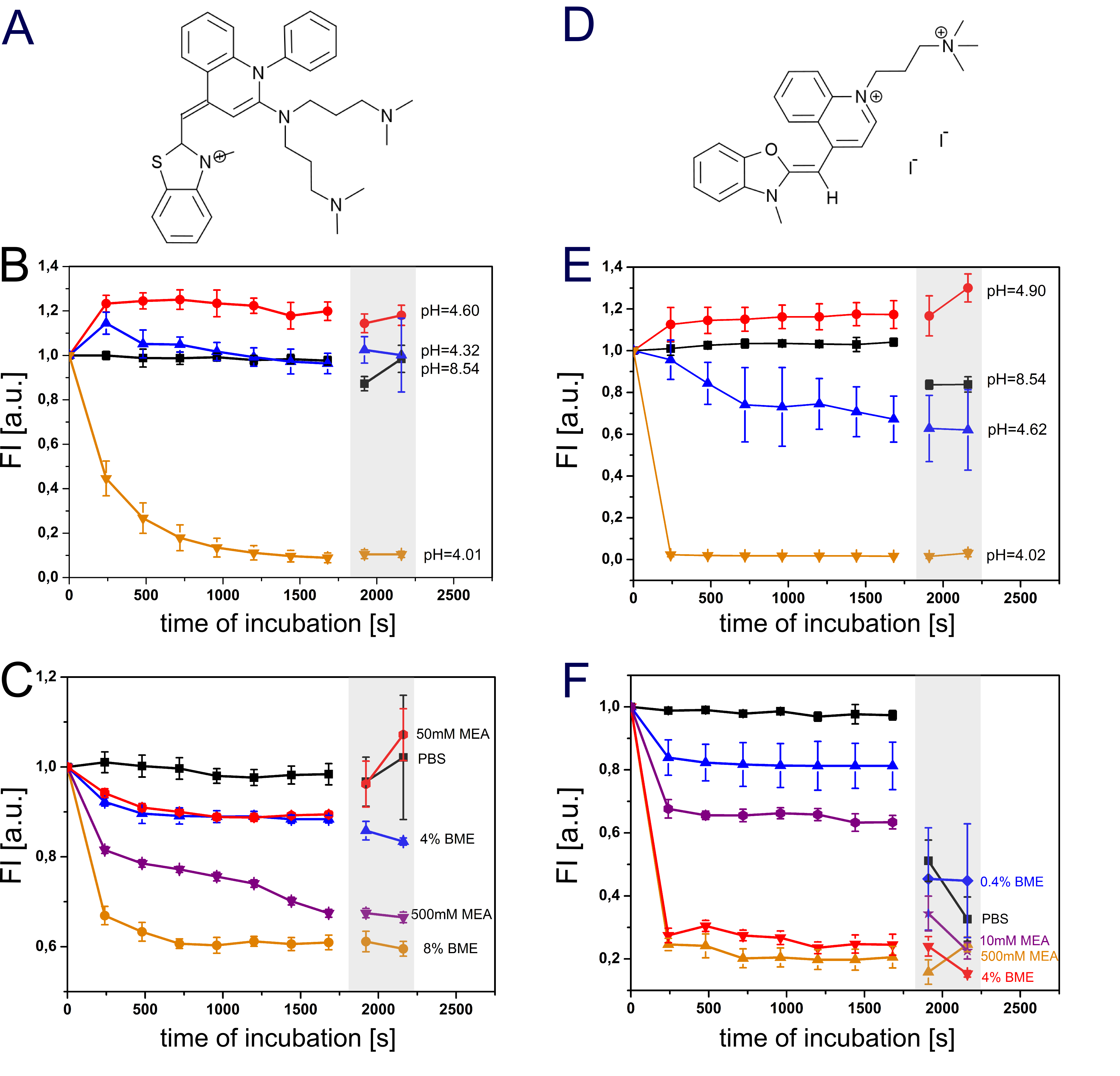


**Supplementary Figure S2.** **Influence of the chemical environment on PicoGreen (1:10,000) and YO-PRO-1 (20 nM) fluorescence signal intensity in cell nuclei studied with confocal microscopy.** (**A, D**) Chemical structures of PicoGreen (A) and YO-PRO-1 (D). (**B, C, E, F**) The effect of acidic pH (B,C), β-mercaptoethylamine (MEA) and β-mercaptoethanol (BME) (E, F) on fluorescence intensity of PicoGreen and YO-PRO-1 (FI). The grey rectangles indicate data points acquired after replacing the buffer with water (B, E) or PBS (C, F). Interestingly, acidic buffers cause a decrease of the fluorescence intensity of PicoGreen, YO-PRO-1 as well as YOYO-1 (showed in **Fig. 1**) more effectively than high concentrations of primary thiols (~5 - 15% of fluorescence signal remained when acidic conditions were applied, as compared to 20 - 60% with thiols). For more details see **Supplementary Note 1**.


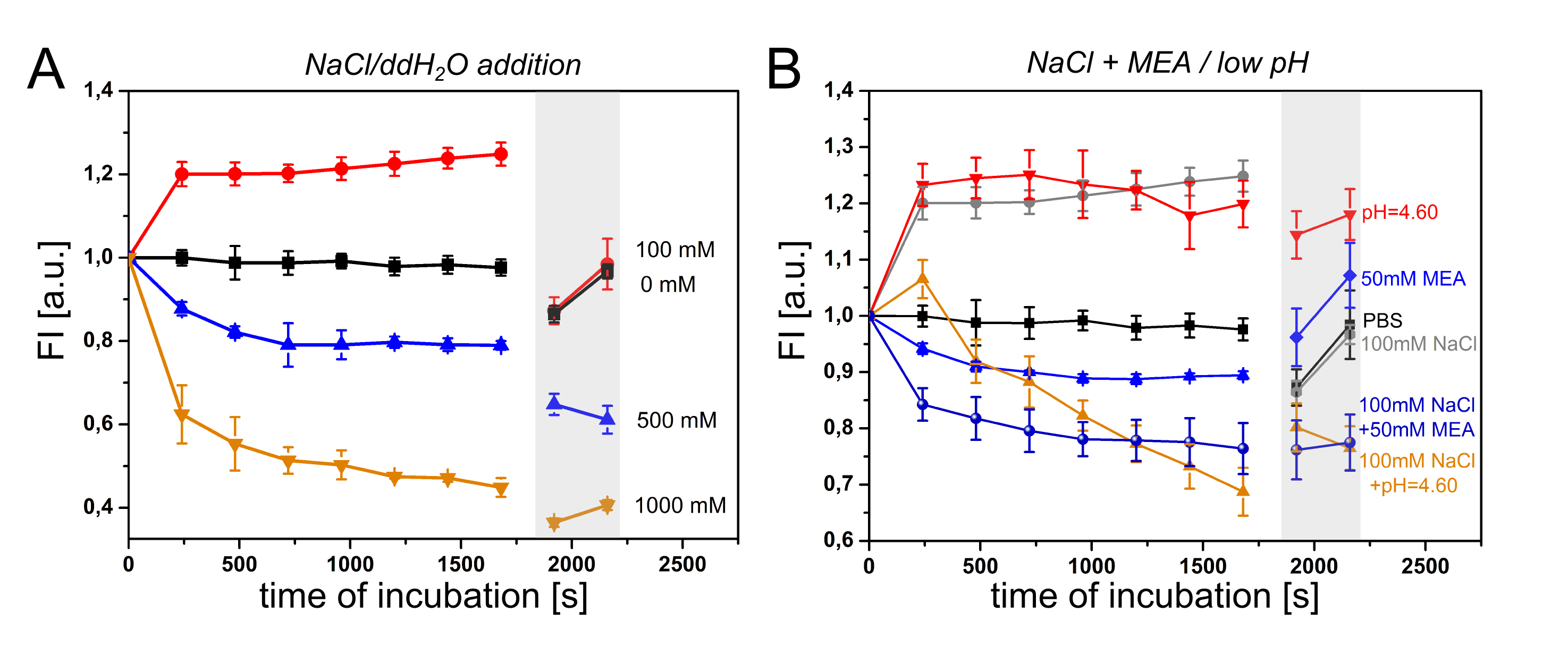


**Supplementary Figure S3. Chemically induced decrease of the fluorescence signal of DNA binding PicoGreen using increased buffer ionic strength.** **A**) Time-lapse confocal microscopy measurements of fluorescence intensity (FI) of PicoGreen bound to the cellular DNA upon addition of NaCl from 0 - 1000 mM concentration range. For high concentrations of NaCl, the PicoGreen signal intensity underwent an immediate decrease that continued for the next 20 min, likely indicating its gradual dissociation from DNA (for more details see **Supp. Note 1**). This indicates that the electrostatic interaction of PicoGreen with dsDNA in fixed cells can be modulated in order to manipulate DNA-binding strength potentially useful in fBALM. **B**) Increased ionic strength in combination with an acidic buffer (orange and red) and with β-mercapthoethylamine (MEA, cyan and blue) is likely to enhance PicoGreen dissociation from the complex with DNA (FI decrease in time). This indicates that dsDNA denaturation is not necessary to release the dye from DNA. Note that in 100 mM NaCl/H_2_O (grey) or low pH equal to 4.60 (red) alone, the PicoGreen signal is rather stable over time. **Supp. Fig. S3B, C** shows that in buffers with low ionic strength, higher concentrations of MEA or lower pH has to be applied in order to affect the interaction of PicoGreen with DNA**.**


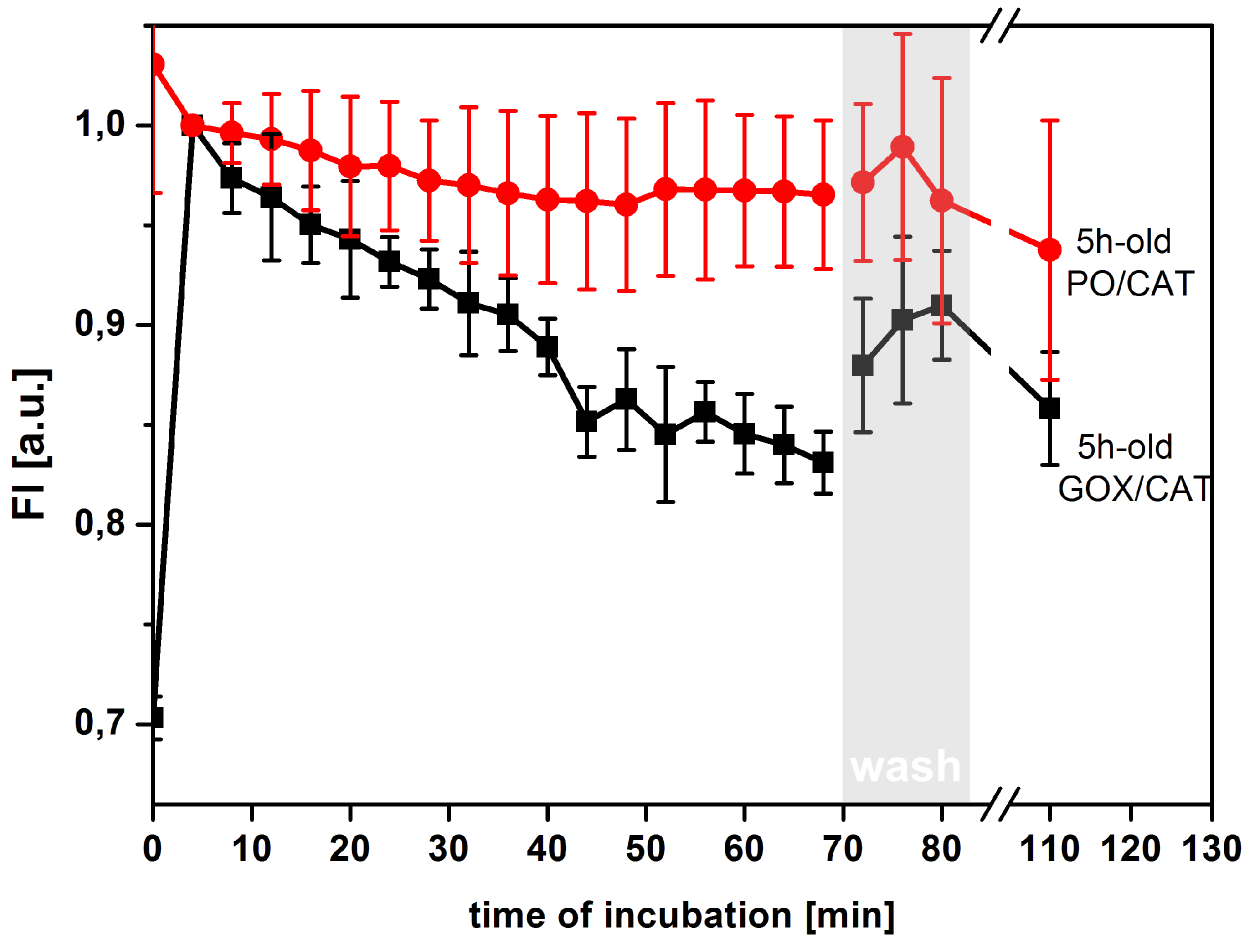


**Supplementary Figure S4. Measurement of YOYO-1 signal intensity over time after addition of 5h-old buffers containing enzymatic oxygen scavenging systems:** GOX/CAT/glucose (black) or PO/CAT/glucose (red), 5h after preparation of these imaging buffers. The first data-point (t=0) was acquired in PBS for both. Subsequently a specific buffer was added and a signal acquisition (t=1,…,N) followed. The experiment was performed in a similar way as experiments in **Fig. 1** and **Supp. Fig. S2, 3**. Here, the data were normalised with respect to the first data-point acquired after addition of oxygen scavenging system (t=1). Grey rectangle indicates the data-points preceded by 4 washing steps with PBS each. The last 4 data-points for each curve were acquired in PBS.

**
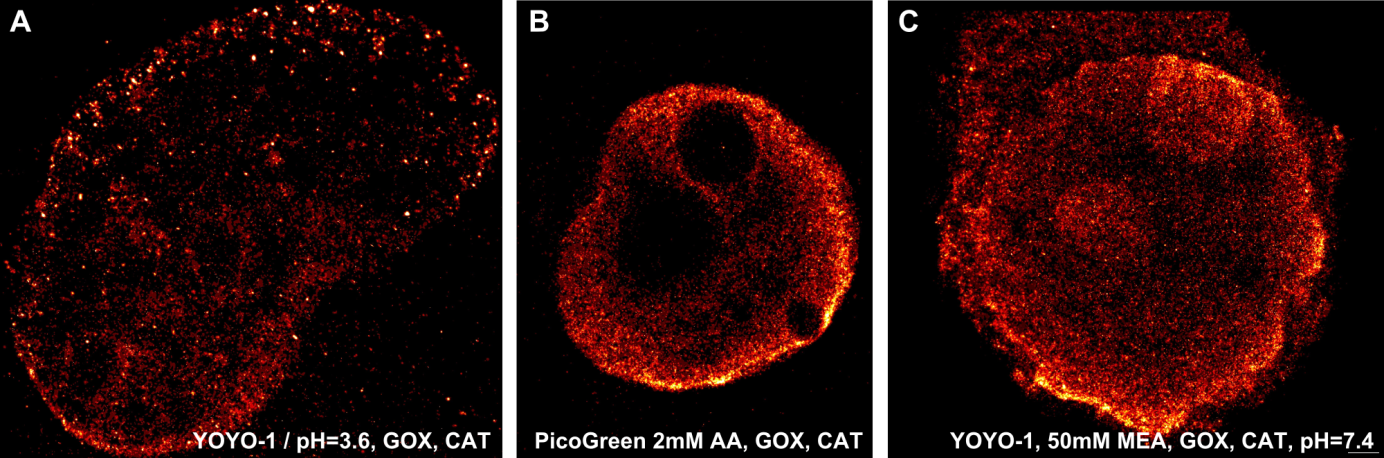
**

**Supplementary Figure S5. SMLM imaging of cells pre-labelled with YOYO-1 or PicoGreen.** A) A HeLa cell nucleus stained with 12 nM YOYO-1, imaged in an hypoxic imaging buffer of pH~3.6. B) Hodgkin’s lymphoma cell pre-labelled with 1:5,000 PicoGreen, imaged in a hypoxic buffer containing 2 mM ascorbic acid. C) Hodgkin’s lymphoma cell pre-labelled with 10 nM YOYO-1, imaged in a hypoxic buffer containing 50 mM MEA. None of these preparation methods outperformed images aquired with pBALM using gradual buffer acidification with GOX presented in **Fig. 4, 5**.


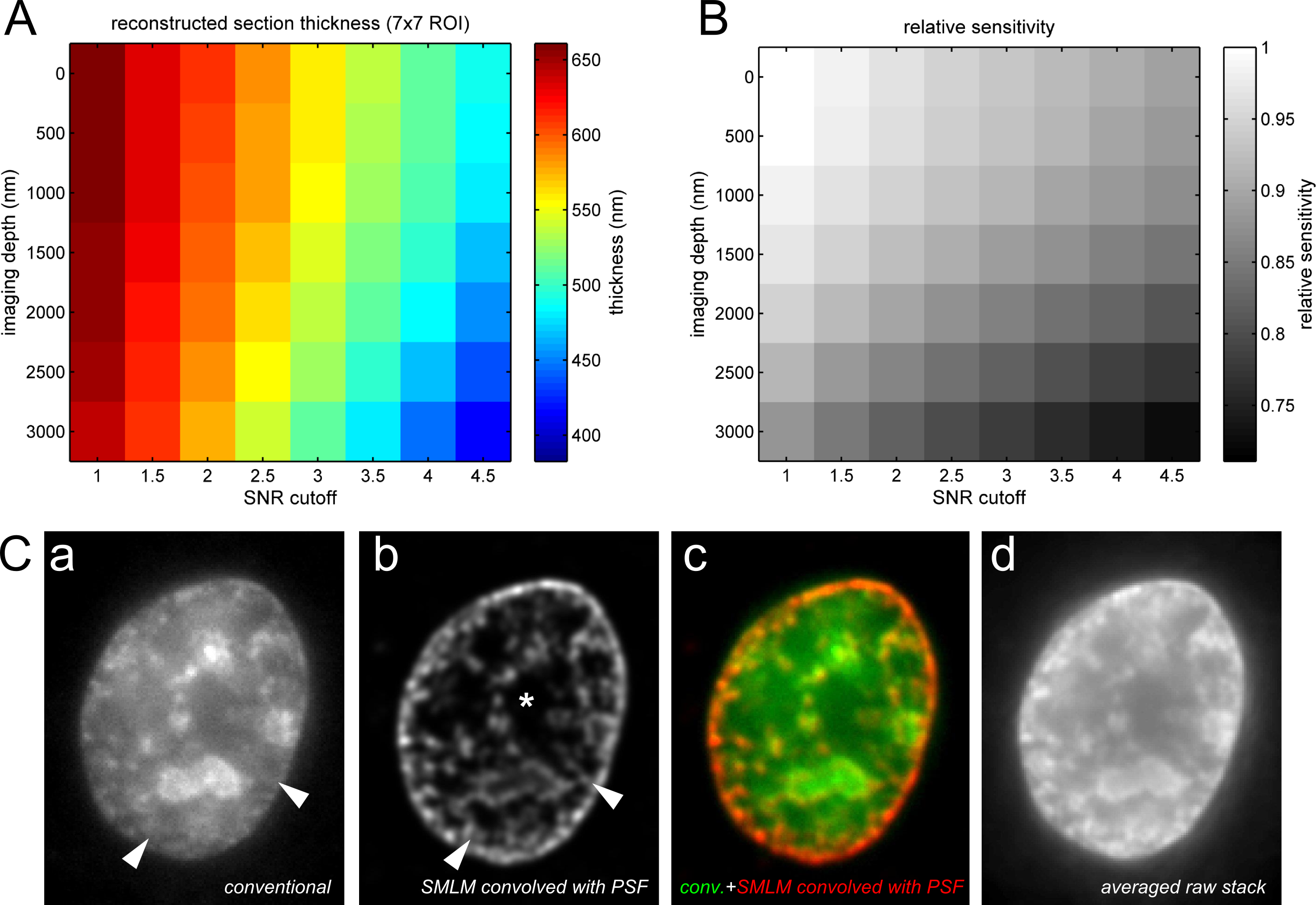


**Supplementary Figure S6. Sectioning capability of fBALM is superior to regular widefield microscopy depth of field. A**) Estimation of the thickness of the reconstructed SMLM images is based on computer simulated SMLM data. Aberrated widefield microscope point-spread-functions (PSF) were calculated for the given refractive indices of oil, cover glass, and imaging buffer (n = 1.3477, i.e. refractive index of water with 10% glycerol). With the help of the computer generated depth-dependent PSFs, diffraction limited signals were generated on top of a background level at various z-positions inside the sample and with a varying z-focus position for detection; the data was subjected to evaluation using the standard software (see Materials and Methods) with free parameter SNR, corresponding to the threshold value applied to detect signals (see Materials and Methods). The z-thickness was then extracted as the z-extent of the simulated signals which were detected by the standard SMLM evaluation procedure. **B**) The relative sensitivity indicates the probability for signal detection in the vicinity of the corresponding imaging depth, normalised to the maximum detection efficiency achieved at the interface between a coverslip and the sample and the lowest applicable threshold values. Note that such low threshold values cannot be applied in practice as we would pick up a lot of false positive signals from the background noise (Gruell et al., 2011). **C**) Simulated widefield images based on the experimental SMLM data (*b*) indicate an optical sectioning capability not inherent to widefield microscopy (*a*). In (*c*) a merged image of (*a*) and (*b*) is presented. SMLM raw data stack has been averaged throughout all frames and presented in (*d*). Asterisk denotes a part of the nucleus where no single molecule events occurred as revealed by fBALM and confirmed by visual inspection of the raw data stack. Arrow-heads indicate differences between images in (*a*) and (*b*). Image in (*b*) was generated from the fBALM list of localizations using Gaussian blurring with standard deviation corresponding to the widefield microscope PSF instead of the localisation precision.


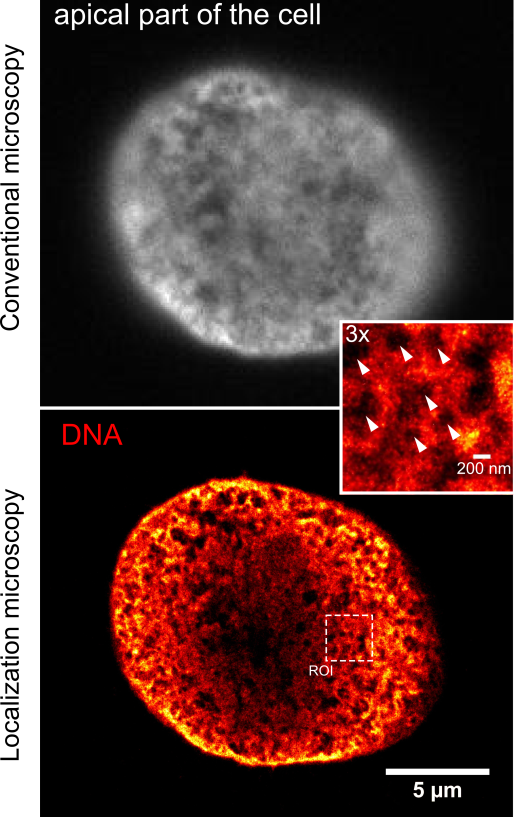

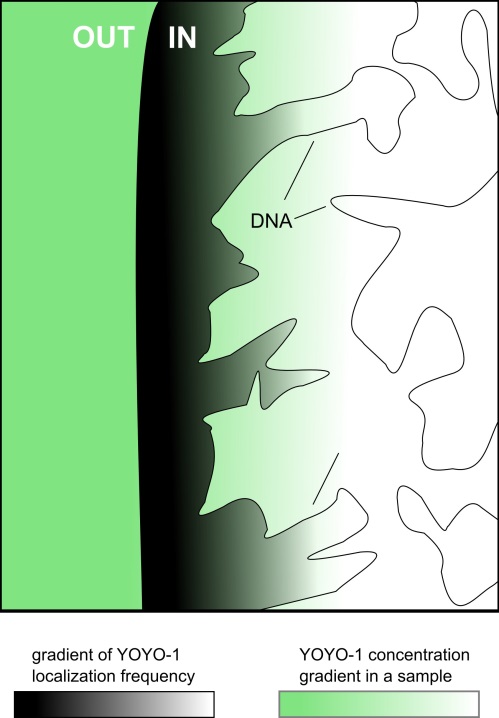


**Supplementary Figure S7.** **DNA structure fluctuation-assisted BALM imaging of the nuclear periphery (bottom) of HL-1 cell using YOYO-1.** Cells were permeabilised with 0.5% TX-100, stained with <10 nM YOYO-1, and submerged in a buffer containing glucose oxidase and catalase. Imaging was performed after 7 h incubation, i.e. when the majority of the dye molecules can be expected to have escaped from the cell nucleus into the imaging buffer. **Left**) The anticipated schematic design of the experimental approach and hypothetical YOYO-1 local concentration occurrence at the cell’s bottom. The vast majority of single molecule localisations were obtained from the periphery, not from the interior, resulting in a high signal to noise ratio for single molecule binding events. **Right**) DNA structure fluctuation-assisted BALM results. The zoom-in inset indicates terminal parts of interchromatin channels (arrow-heads) connecting the interior of the cell nucleus with the cytoplasm through a nuclear pore complex (Schermelleh et al., 2008; Cremer et al., 2015;). For comparison, the conventional widefield image is presented in grey.


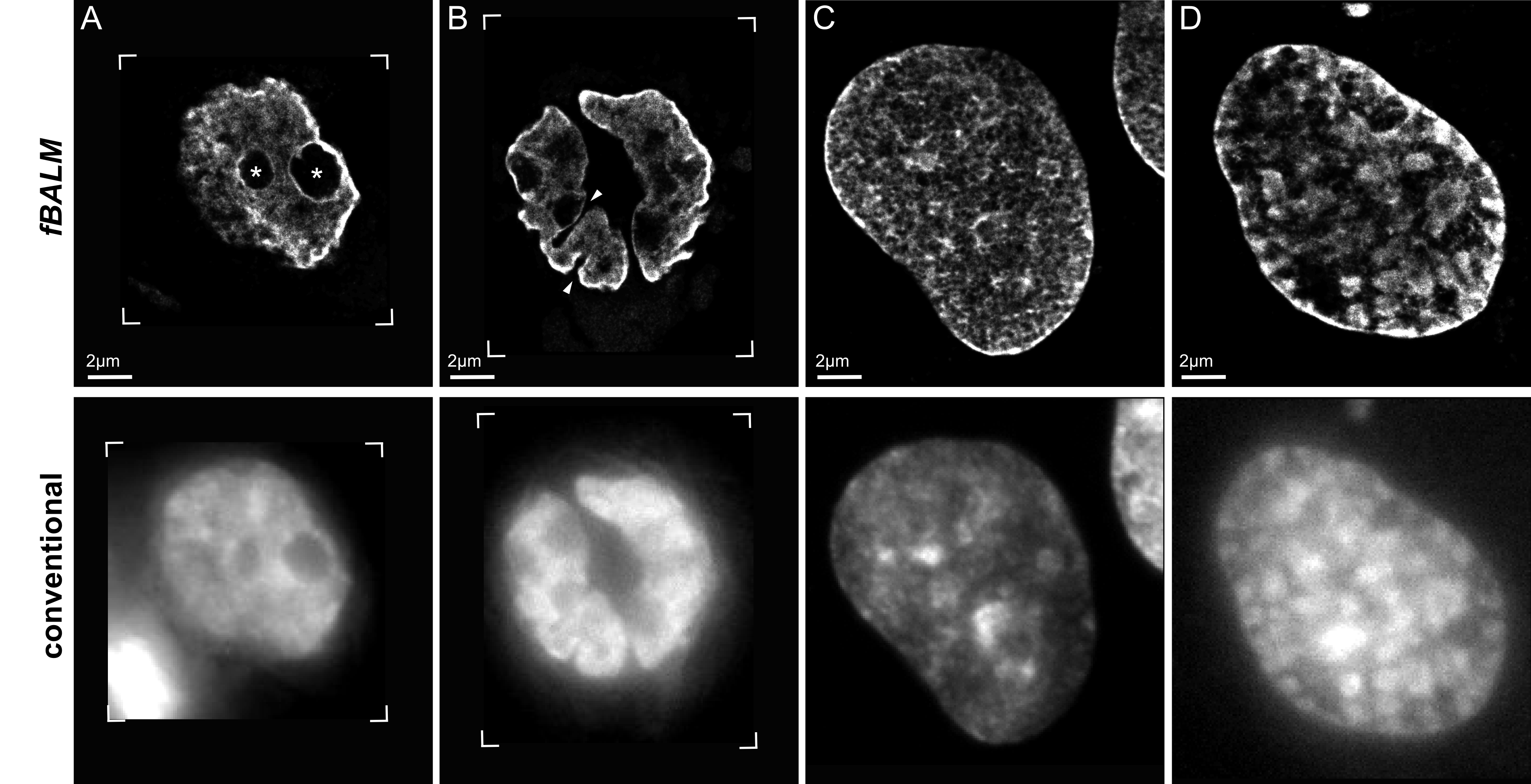


**Supplementary Figure S8.** **Super-resolution fBALM imaging with YOYO-1 of various cell types helps to discriminate differences in their nuclear architecture.** Mononucleated Hodgkin’s lymphoma (**A**) and binucleated Reed-Sternberg (**B**) cells found in HDLM-2 culture. Nuclear envelope invaginations are indicated with white arrowheads. Characteristic „holes” (A) are indicated with white asterisks (Righolt et al., 2014). **C**) HL-1 cardiomyocyte presented for comparison. **D**) A neuroblastoma cell from a Neu-2 culture. All cells were imaged after approx. 4h incubation with a buffer, undergoing slow pH decrease (for details see **Fig. 3**). White lines indicate corners of the imaged area.

**
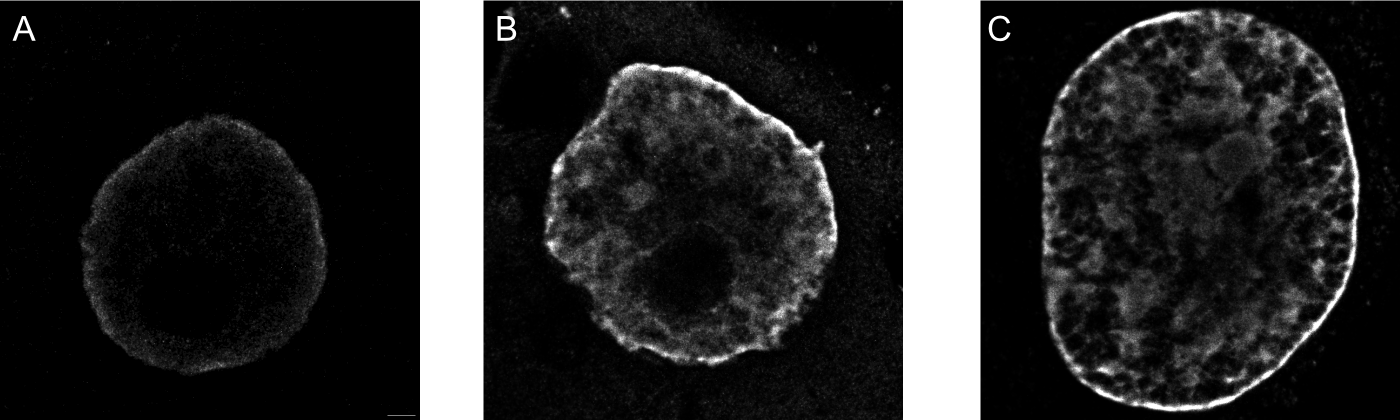
**

**Supplementary Figure S9.** **Influence of laser intensity, fixation protocol and nuclear membrane permeabilisation on fBALM imaging of Neuro-2a cells.** **A**) Cell fixed for 15 min with formaldehyde and permeabilised for 20 min with 0.5% TX-100. The cell was imaged with high laser power (90 mW). Most YOYO-1 signals locate at the nuclear periphery; this can be attributed to limited penetration of the dye into the nucleus. For more details see **Supp. Note 3**. **B**) Cell processed in the same way as in (A) but imaged with two times lower intensity of the excitation light. **C**) Cell fixed using ice-cold methanol on ice for 10 min, permeabilised using 0.1% TX-100 and exposed to a low excitation intensity. All cells were labelled with YOYO-1 for ~45 min prior the fBALM measurement.


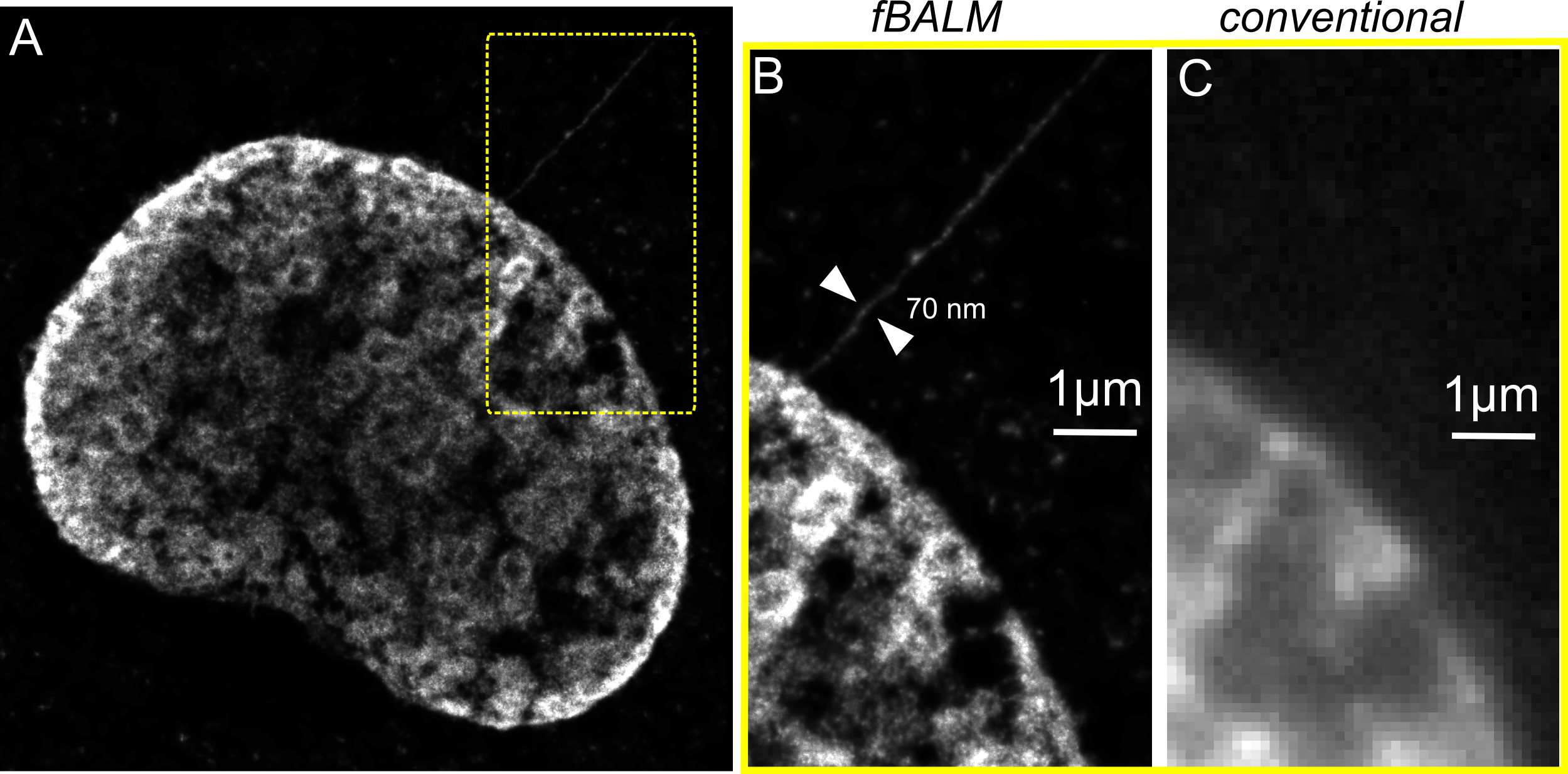


**Supplementary Figure S10. Demonstration of fBALM DNA specificity and contrast on a chromatin bridge in a cardiomyocyte cell subject to ischemic treatment.** **A**) Image of the entire cell nucleus. Yellow rectangle indicates a region of interest further enlarged 2x in (**B, C**). A chromatin bridge is clearly visible when imaged with fBALM (**B**), unlike in the conventional fluorescence widefield image (**C**) where few YOYO-1 molecules bound to this chromatin structure are not sufficient to detect it. Chromatin bridges often form as a cause of failure in chromosome segregation in different aberrant cancer cell lines. Small signal clusters in the cytoplasm likely correspond to mitochondrial DNA.


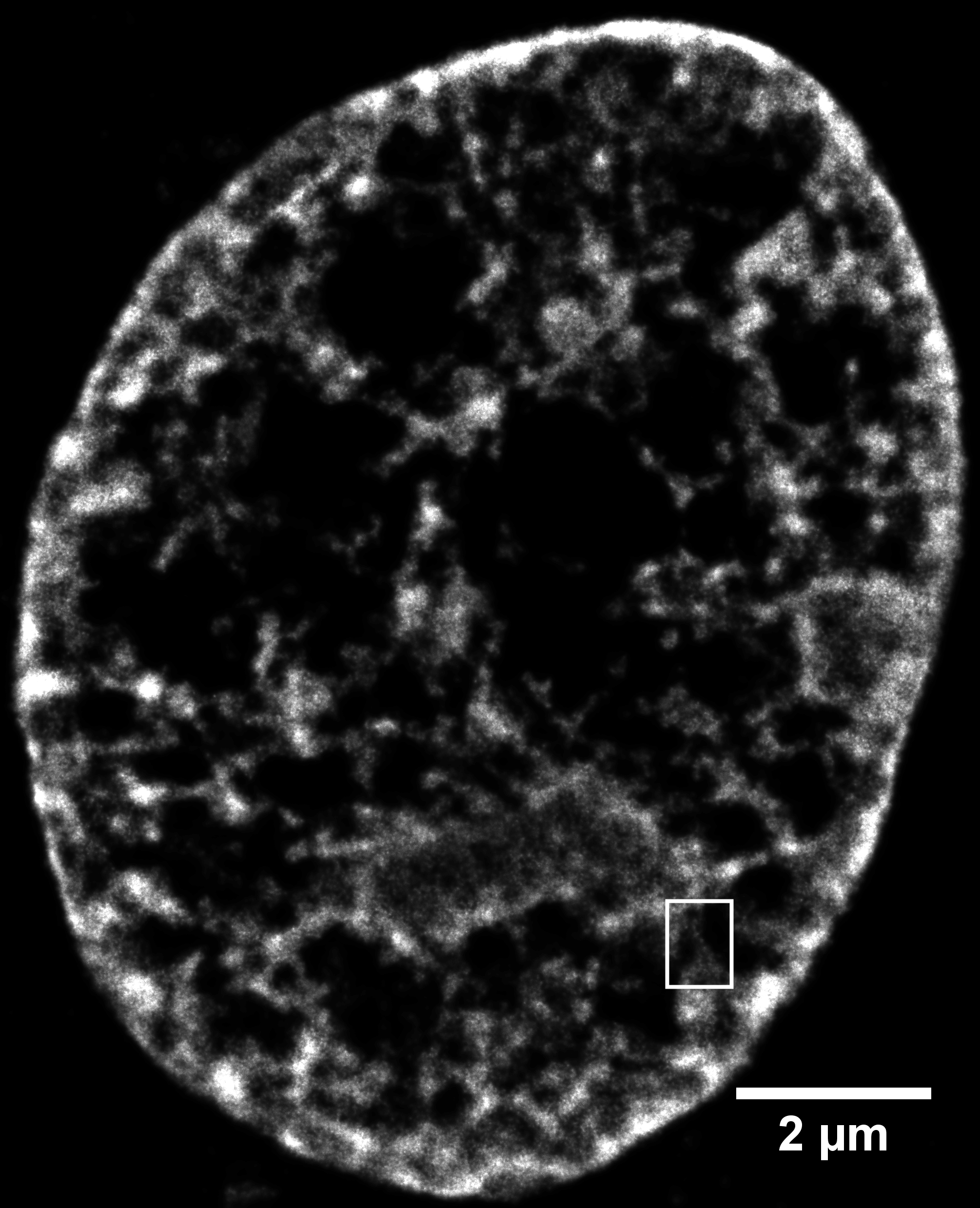


**Supplementary Figure S11. Signal count for the observed chromatin clusters.** Reconstructions of fBALM data from **Fig. 5** are indicative of chromatin clusters that can be observed in the zoomed-in version of the region of interest (white rectangle). The signal clusters defined by 50% of the maximum intensity of the zoomed-in version are highlighted in green. The number of signals counted in the outlined areas are given, together with the number of signals in "void" regions (marked in blue). Scale bars: 2 µm (overview) and 100 nm (zoomed in version).

|  | **Extinction coefficient at exc. max.**  **[cm^-1^M^-1^]** | **dsDNA Binding constant**  **[M^-1^]** | **Quantum efficiency while bound to dsDNA** | **Peak absorption/ emission with dsDNA**  **[nm]** | **Fluorescence fold increase and binding mechanism** |
| --- | --- | --- | --- | --- | --- |
| **Hoechst 33258** | **47,000** | **10^6^-10^7^** (Drobyshev et al., 1999) | **0.42**  (Cosa et al., 2001) | **349/466** (Cosa et al., 2001) | **13x**, **mgb** (Cosa et al., 2001) |
| **DAPI** | **27,000** | **10^5^-10^6^**  (Manzini et al., 1985) | **0.34**  (Cosa et al., 2001) | **358/456**  (Cosa et al., 2001) | **18x**, **mgb**  (Cosa et al., 2001) |
| **YOYO-1** | **98,900**  (Benvin et al., 2008) | **10^10^-10^12^**  (Glazer and Rye, 1992) | **0.38** | **458/489** (Rye et al., 1992) | **<1,000x**  (Cosa et al., 2001; Rye et al., 1992), **i** |
| **YO-PRO-1** | **52,000** (Benvin et al., 2008) | **10^5^-10^6^**  (Glazer and Rye, 1992; Joseph et al., 1998) | **0.44** | **475/486** (Rye et al., 1992) | **700x**  (Rye et al., 1992),  **i** |
| **PicoGreen** | **70,000** | **5x10^9^**  (Dragan et al., 2010) | **0.5** | **498/528** (Cosa et al., 2001) | **1,070x**  (Cosa et al., 2001), **mgb**, **i** (Dragan et al., 2010) |
| **Ethidium bromide** | **~6,000** | **~10^5^**  (Alonso et al., 2006; Vardevanyan et al., 2003) | **0.35**  (Cosa et al., 2001) | **520/610**  (Rye et al., 1992) | **35x**  (Rye et al., 1992),  **i** |
| **SybrGreen** | **70,000** (Dragan et al., 2012) | **10^7^**  (Dragan et al., 2012) | **0.42** (Dragan et al., 2012) | **~500/525** | **>1000x**, **mgb** (Dragan et al., 2012) |
| **DRAQ5** | **20,949** (Smith et al., 2000) | **-** | **0.004**  (Njoh et al., 2006) | **646/681** (Smith et al., 2000) | **~1x**  (Njoh et al., 2006), **i**  (Smith et al., 2000) |

**Supplementary Table 1. Overview of physical properties of DNA-binding dyes.** From the table one infers that cyanine-derived dyes (in particular YOYO-1 and PicoGreen) have the highest photon emission. Among the brightest dyes listed here, YO-PRO-1 and PicoGreen bind to dsDNA most weakely. YOYO-1 is the brightest among all DNA binding probes reviewed here. The binding mechanism indicated as ‘i’ – intercalation or ‘mgb’ as minor groove binding. ‘–‘ information not found. A lack of reference indicates that the information was obtained from the provider’s website.

| **Labelling method**  **Feature** | **DNA dyes** | **α-core-histone immunofluorescent labelling** | **5-ethynyl-2’-deoxyuridine long-term incorporation followed by click-it** |
| --- | --- | --- | --- |
| **Best achievable labelling density**  **Additional localisation errors**  **Chromatin labelling specificity**  **Molecular weight of a probe and its chromatin penetration**  **Other limitations**  **Other advantages** | Every ~3.2 bp  for YOYO-1  (Günther et al., 2010)  Direct labelling of the DNA results in no steric localisation error  High affinity to double stranded DNA, no unspecific fluorescence background  0.6 – 1.5 kDa,  moderate penetration issues for heavier DNA dyes e.g. YOYO-1,  good penetration for e.g.  YO-PRO-1  (**Supp. Fig. S1**)  Sequence specificity  [for example see  (Reisner et al., 2010)], fixed dipole once bound to the DNA leading to signal asymmetry and to nanometre localisation error (Mortensen et al., 2010; Backer et al., 2016)  Fast labelling | Every 146 + (10 – 50) bp corresponding to one nucleosome + linker DNA, possibly less for nucleosome free regions  Additional steric localisation error of approx. 15 - 20 nm from 1^o^ and 2^o^ antibody size (Ries et al., 2012)  Moderate epitope recognition specificity due to steric hindrance, fluorescence non-specific background present  ~150 kDa (IgG),  strong penetration issues (Bancaud et al., 2009; Pierzyńska-Mach et al., 2016)  Multiple blinking of switchable fluorescent probes  Possibility to use feasibly photoswitchable synthetic carbocyanine- or rhodamine- derivatives, fluorophore dipole effects negligible (Vaughan et al., 2012) | Difficult to predict  Additional small steric localisation error of approx. 1 nm  Click-it has a typical reaction yield of ~90%  (Wang et al., 2003; Himo et al., 2005), unknown DNA incorporation rate  ~1.3 kDa (Alexa647),  Penetrations issues have not been tested, likely similar to DNA dyes  EdU treatment induces DNA repair signalling and blocks cells in G2 cell cycle phase (Zhao et al., 2013), likely influencing chromatin structure, cannot be used with samples from patients, multiple blinking of switchable fluorescent probes, sequence specificity  Possibility to use feasibly photoswitchable synthetic carbocyanine- or rhodamine derivatives |

**Supplementary Table 2. A comparison between chromatin labelling strategies for super-resolution SMLM.** The DNA dyes can be used as proposed in this manuscript, based on the original concept of (Schoen et al., 2011). Anti H3 and H2A or H2B immunofluorescence labelling for SMLM has been originally proposed by (Ricci et al., 2015). Long-term EdU incubation of cells throughout an entire cell cycle phase followed by fixation and click-it reaction with a switchable fluorophore was put forward by (Zessin et al., 2012).

**Extended Materials and Methods**

*Confocal Microscopy*

For fluorescence intensity measurements of dye association and putative dissociation in time in entire cell nuclei we used an inverted Leica SP5 Confocal microscope equipped with 63x/1.4 NA objective lens and a Hybrid detector suited for photon counting (Leica Microsystems, Germany). The 488 nm line of an argon laser was used and fluorescence from YOYO-1, YOPRO-1 and PicoGreen (all purchased from Lifetechnologies) stained cells was detected in the 490 - 560 nm emission range; 512 x 512 pixel images from 5 - 6 fields of view each were collected. The pixel size (object plane equivalent) corresponded to 303 nm. Measurements were performed on 3.7% formaldehyde fixed (for 10 min) and 0.5% Triton X-100 (for 10 min) treated HeLa cells seeded on 500 µl µ-slide 8-well chambers with a microscopic-glass bottom (IBIDI, Germany). For dye dissociation studies cells were stained with 12 nM YOYO-1 for 1h, 1:10,000 PicoGreen for 40 min, or 30 nM YO-PRO-1 for 40 min (500 µl volume). Cells were washed with PBS overnight. For dye association studies, an initial 'background' image was acquired in 250 µl of PBS (Gibco) and was followed by the addition of 250 µl of 2x concentrated dye solution. Images were acquired every 2 min. For the dye dissociation assay we used the same method with a few exceptions: for studies on pH dependence, an initial image was performed in distilled water that was replaced thereafter with a buffer of a given pH; these images were acquired every 4 min. First 8 data points were followed twice by 4 washing steps (using 750 µl PBS or H_2_O each) and a signal acquisition. All samples were treated with 0.5 U/ml RNase A and 20 U/ml RNase T1 (Ambion, USA) for 1 h at 37 °C, to diminish an effect of RNA-associated dye fluorescence.

For investigation of gross changes of chromatin structure, 3D confocal microscopy (Leica SP5) followed by deconvolution was used (SVI 3D Huygens Deconvolution & Analysis Software (Scientific Volume Imaging B.V., Hilversum, Netherlands)). HeLa cells were grown for two days on 8-well µ-slide (IBIDI, Germany), incubated for 12 min with 10 µM 5-ethynyl-2’-deoxyuridine (EdU), then fixed with 3.7% formaldehyde and permeabilised with 0.5% TX-100 (Sigma-Aldrich, Germany). Then a “click-it reaction” with Alexa 555 was carried out according to manufacturer's instructions (Lifetechnologies). This procedure enabled the 3D imaging of chromatin-bound reference points in the same cells (due to DNA replication sites) before and after a hypothetical structural change. Confocal images with 60 nm object plane pixel size (x, y) were acquired with a z-step (along the optical axis) of 130 nm. Local maxima were found using an ImageJ plugin 3D Foci Picker calculating centres of fluorescence gravity. Each data stack was separately evaluated and visually inspected for the quality of maxima hits. Nearest neighbour (nn) distance analysis was performed using MATLAB (Mathworks). 5 cells were evaluated per condition before and after subjecting them to the acidic treatment. The difference in mean nearest neighbour distance was calculated for each cell separately by subtracting the mean value after treatment from the respective mean value of the same cell prior to the treatment. In this way the outcome of a negative value reflects shrinkage whereas the positive one reflects expansion of the nuclear structure analysed. Nuclear volumes were calculated using convex-hull determination based on foci 3D coordinates in MATLAB. Changes in the cell nuclear volume (Δ) were calculated using the following formula: Δ=1-V_before_/V_after_, where V corresponds to the estimated volume.

*Single Molecule Localisation Microscopy*

Sample preparation: Cells of a HL-1 murine cardiac muscle cell line (Claycomb et al., 1998) were grown on 20 x 20 x 0.16 mm^3^ gelatine/fibronectin-coated coverslips (Menzel-Gleaser, Germany), were fixed (see below) and stained, typically using 10 - 12 nM YOYO-1 for 45 min. For DNA structure fluctuation-assisted BALM (fBALM) we used a PBS based imaging buffer (Gibco) containing 0.5 mg/ml glucose oxidase (GOX), 40 µg/ml catalase and 10% (w/v) glucose (initial pH=7.6). As a control we used another oxygen scavenging system known to maintain a stable pH in time (Swoboda et al., 2012), based on 5 U/ml pyranose oxidase, 57 µg/ml catalase and 10% (w/v) glucose (all purchased from Sigma-Aldrich). A coverslip was immersed in an imaging buffer, embedded in ~25 µl of the same imaging buffer and fixed to the glass slide using a rubber cement. Sealing the sample tightly using Picodent Twinsil® prevented oxygen influx from the ambient air.

For the investigation of changes of chromatin structure in ischemia, live HL-1 cells were placed in a hypoxia chamber (Whitley Hypoxystation H35) with 1% O_2_ and 70 - 85% humidity. The ischemic environment was introduced for 1 h using a pre-equilibrated buffer containing 115 mM NaCl, 12 mM KCl, 1.2 mM MgCl_2_, 2 mM CaCl_2_, 25 mM HEPES and 5 mM deoxyglucose. This preparation protocol was previously validated and resulted in 9-fold transcription rate downregulation and a ~7 fold decrease in ATP levels (Kirmes et al., 2015). Afterwards the cells were fixed with ice-cold methanol for 10 min on ice and permeabilised for 10 min with 0.3% TX-100. We used methanol-based fixation, as previously the DNA was reported to be slightly more resistant to denaturation after formaldehyde treatment (Traganos et al., 1975).

For the SMLM studies reported in **Fig. 4E,** HL-1 cells were treated for 10 min with 10 µM EdU prior to 3.7% formaldehyde fixation and labelled by means of the “click-it” reaction with Alexa 488, according to the protocol of the manufacturer (Life Technologies). Measurements were performed using ProlongGold as a mounting medium, as in our hands this medium reduces the extent of multiple blinking and increases Alexa 488 brightness per molecule. Anti-H3 immunostaining with Alexa 647 was performed as previously described (Kirmes et al., 2015), and an imaging buffer containing 50 mM MEA as a switching agent was employed in the absence of oxygen.

Hodgkin’s lymphoma cell line HDLM-2 was prepared for microscopy as previously reported, with a few modifications (Righolt et al., 2014). After formaldehyde fixation cells were attached on poly-L-lysine coated coverslips, permeabilised with 0.1% TX-100 for 10 min, RNase (Ambion, USA) treated in 37^o^C overnight and stained with 12 nM YOYO-1 for 1h. Neuroblastoma Neuro-2a (ATCC® CL-131™) cells (kindly provided by Dr. V. Tiwari, IMB Mainz) were cultured in 37^o^C and high humidity in a Dulbecco’s modified Eagle medium supplemented with glutamine, nonessential amino acids, and 10% fetal bovine serum (Gibco, Germany). Cells were either fixed with 3.7% formaldehyde or with ice-cold methanol and permeabilised using 0.1 or 0.3% TX-100. Cells were stained for 1.5h with 15 nM YOYO-1.

Measurements: Super-resolution experiments were performed on a custom built widefield microscope described in detail previously (Szczurek et al., 2014). Briefly, the microscope was equipped with an air-cooled 12 bit CCD camera (SensiCam QE, PCO imaging) and a 63x, 1.4 NA oil immersed objective. The effective pixel size (object plane equivalent) was 102 nm. A 491 nm diode laser was used for excitation (Calypso 05 series, Cobolt, Sweden) with an intensity in the sample plane of 1 - 2 kW/cm^2^ (collimated laser). Single fluorophore signals were captured after passing through a 525/50 nm bandpass filter (Chroma Technology Corp.). 30,000 frames with an integration time of 20 ms each were acquired and saved as 16-bit .tiff stacks with the highest 4 bit set to zero. Prior to the measurement 3 conventional widefield images were acquired with 120 ms integration time and were averaged for presentation. The number of frames chosen provided optimal quality reconstructions despite the fact that single molecule events occur for even longer than 2 – 3 h. Longer measurement times, however, result in progressive photobleaching of YOYO-1 within the nucleus and increased contribution of signals originating from the periphery (for details see **Supp. Note 4**). For EdU-Alexa 488 labelled cells, 3,000 - 5,000 frames with 50 ms camera integration time were acquired. For H3-Alexa 647, 25,000 frames with 25 ms camera integration time were acquired; ~2 kW/cm^2^ 647 nm diode laser intensity was used for excitation (LuxX, Omicron, Germany).

Data analysis: We used a fastSPDM updated MATLAB software (https://code.iri.uni-frankfurt.de/trac/microscopy) (Gruell et al., 2011), as in our hands it performs better on noisy data with conceivable out-of-focus background (the typical situation encountered in SMLM imaging of 3D intact cell nuclei). Briefly, an initial background image is estimated as the average of the first 8 frames. After subtraction of a background image, only individual molecule signals with a peak intensity exceeding 3x noise standard deviation were subjected to further analysis (Poisson noise distribution model assumed for the acquired images). The background image was then refined for analysis of the subsequent frames as follows: in the processed 2D image, detected signals were clipped; the resulting image was averaged with the previous background image to yield the new adaptive background estimate. This procedure diminishes the bias in single molecule event detection of signals in an area with elevated local background; however, signals in areas with high background (and therefore higher noise in the background) will be affected by higher fluctuations in their peak intensity. For further details on the algorithm, see (Kirmes et al., 2015). From the list of localisations, approximately 30% were discarded routinely from the analysis based on their large PSF width. This provides elimination of overlapping signals and strong out-of-focus signals which otherwise would adversely affect the final image resolution (Burgert et al., 2015). The algorithm also merged single molecule signals appearing in subsequent frames, using a search radius of 2x average localisation precision (typically around 20 nm). To correct for mechanical drift during the measurement, we implemented a cross-correlation analysis between sample reconstructed images (typically 1,000 frames each), and drift vectors were extracted. After interpolation the list of single molecule x and y positions was altered according to the frame index. The correction resulted in a standard deviation of less than 5 nm between sample images in the corrected dataset. In order to estimate the structural resolution in the SMLM images, Fourier ring correlation (FRC) analysis was performed utilising a custom written MATLAB program similar to the method described in (Banterle et al., 2013; Nieuwenhuizen et al., 2013). First, the localisation dataset was randomly split into halves, and next Fourier analysis was carried out for each of subsets separately. Furthermore, the correlation between their Fourier images was performed within concentric rings radially expanding from the 0^th^ frequency. A 1/7 experimental cut-off (Nieuwenhuizen et al., 2013) was used to read out the FRC resolution from the intersection with FRC polynomial fit (25^th^ order).

For the chromatin density distribution analysis we binned the single molecule data in a 60 x 60 nm^2^ grid using a hist3 MATLAB function. Histograms of the number of single molecules per square bin and their cumulative probability were calculated using Origin 8.6 software (OriginLab). We also investigated the grid size in a range from 20 to 100 nm and they all yielded similar results.

Simulated data: Estimation of the sectioning capability of the SMLM approach realised in fBALM was obtained from analysis of computer generated data. Using the refractive indices of oil, cover glass, and imaging buffer (n = 1.3477, i.e. refractive index of water with 10% glucose), widefield microscope point-spread-functions (PSFs) with spherical aberrations were calculated for imaging wavelength 520 nm at various imaging depths. Signals were generated on top of a background level at various z-positions inside the sample and with a varying z-focus position for detection. The signal and background levels were taken as the mean levels from the experimental data. Random noise was added to the data following a Poisson noise model. The computer generated data were then evaluated using the standard software (see above) with free parameter signal-to-noise ratio (SNR), corresponding to the threshold value applied to detect signals. As indicated above in the evaluation of our experimental data, the SNR level was set to a value of 3. The z-section indicated in **Supplementary** **Figure S6** is given as the width beyond which detection probability drops below 50%.

Visualisation: Gaussian rendering of SMLM data may lead to 1.41x loss in image optical resolution (Baddeley et al., 2010); thus a density map approach was chosen. Similar to the visualisation based on Delaunay triangulation, we implemented a Matlab routine that generates “Wigner-Seitz cells” around each detected signal where the intensity of the respective “cell” is set to a value inversely proportional to its area (Baddeley et al., 2010). Finally, the positions of single molecule signals were randomly jittered 100 times with the respective localisation precision and jointly plotted in a final reconstruction with a pixel size corresponding to 5 nm. This procedure smoothens the image and hence helps to perceive structures; however it is less prone to generation of artefacts, and maintains linear proportion between image intensity and localisation density (Baddeley et al., 2010). Basically the same structures were observed using a visualisation approach based on histogram binning similar to the one applied in (Ricci et al., 2015), including profiles (**Fig. 4C**). Software for visualization is available from <https://gitlab.com/microscopy/visualization>.

**Supplementary Note 1 - Monomeric cyanine PicoGreen and YO-PRO-1 DNA dyes and their potential in fBALM: effects of low pH, primary thiols, and increased ionic strength.**

We tested if similar principles identified for YOYO-1 (**Fig. 1**) are applicable to other DNA-binding dyes including monomeric-cyanine intercalator YO-PRO-1 and minor-groove binding PicoGreen. The results of time-course fluorescence measurements for acidic conditions for PicoGreen and YO-PRO-1 strongly resembled the behaviour of YOYO-1. We noted a sharp fluorescence drop for a transition between following pH values: PicoGreen, 4.32 to 4.01; YO-PRO-1; 4.9 to 4.62, i.e. values close to pK_a_ values of hydrogen donors in DNA hydrogen bonds equal to 3.9 and 4.2 (Verdolino et al., 2008) and known for DNA ionisation (Zimmer et al., 1968; Hermann and Fredericq, 1977). It has been previously shown that cyanine DNA-binding dyes have negligible affinity to denatured DNA (Reisner et al., 2010) and that they associate to the DNA immediately upon ssDNA hybridisation (Chen et al., 2013). Thus we attribute the signal loss in our experiment (at least in part) to dye release from a complex with DNA undergoing denaturation. An ease to weaken the interaction with dsDNA for PicoGreen and YO-PRO-1 may be explained by the fact that these are monomeric cyanine dyes (unlike homodimeric, bis-intercalating YOYO-1, **Fig. 3D**) and their binding affinity is significantly lower to begin with (K_mono_ = 10^5^ - 10^6^ M^-1^ as compared to K_bis_ = 10^10^ - 10^12^ M^-1^) (Glazer and Rye, 1992; Günther et al., 2010) (for details see **Supp. Table 1**). In addition, we found that in case of YO-PRO-1, washing steps with PBS alone lead to a signal loss.

In contrast to the results obtained for YOYO-1 (**Fig. 1A**), β-mercaptoethanol (BME) induced a sharp signal drop for both PicoGreen and YO-PRO-1. In case of β-mercaptoethylamine (MEA), already 10x lower concentrations were required to have similar effect on their signal as compared to YOYO-1 (**Fig. 1A**). Noteworthy, in all abovementioned conditions tested we failed to restore fluorescence signal with washing steps (**Supp. Fig. S2E, F**) what could be an indication of (i) dissociation from the DNA or (ii) long-lived light induced dark state formation. We do not understand however why PicoGreen and YO-PRO-1 would be more prone to a dark state formation than YOYO-1 (e.g. YOYO-1 is made from two YO-PRO-1 subunits). Taking into account that YO-PRO-1 can even be washed away from the DNA to some extent using water (~20%) or PBS (~50%) alone, we are more inclined to indicate release from the DNA as a more probable cause of signal decrease (**Supp. Fig. S2F**).

Electrostatic interaction of positively charged DNA-binding dyes has been previously indicated as one of the main driving forces in association of DNA-binding dyes to the DNA (Dragan et al., 2010). For instance an increased ionic strength of a buffer was shown to reduce the dsDNA association constant of PicoGreen (Dragan et al., 2010). We decided to assess this effect in our experimental settings and confirmed its effect for a whole range of NaCl concentrations for cells pre-loaded with PicoGreen (**Supp. Fig. S3A**). This experiment revealed that physiological concentration (100 mM) immediately elevated DNA-bound PicoGreen fluorescence signal by ~20%. Such moderate salt concentration could partially neutralise a repulsion between negatively charged DNA strands making the dsDNA structure stable (Sorokin et al., 1986), consequently, enhancing stability of the excited state of PicoGreen. Such salt concentration was previously demonstrated to promote DNA strands’ hybridisation (Chen et al., 2013). In contrast, NaCl concentrations of 500 mM and more resulted in a ~40 – 60 % loss of PicoGreen signal that could not be restored when buffers were replaced with H_2_O. Due to the fact that abundant dark state formation of PicoGreen in the presence of NaCl is less likely we attribute this signal loss to a dissociation from the DNA.

Furthermore, we found that elevated ionic strength of a medium has an additive effect together with pH and MEA; physiological NaCl concentration strengthens their effect on fluorescence decrease (**Supp. Fig. S3B**). Although NaCl may introduce additional complexity if taken into account in fBALM, concentrations around ~100 mM might be used to tune the dynamics of hybridisation as well single molecule fluorescence output.

**Supplementary Note 2 - Resolution in fBALM**

The general structural image resolution in SMLM is known to be dependent not only on the precision of localisation of a single emitter (proportional to $N^{-\frac{1}{2}}$, where N is the number of detected photons) but also on the density of single molecule detections in the image. Here, the latter can be freely provided by an extension of the measurement time, resulting in a capture of a sufficient number of binding events. Localisation density in our fBALM images reached on average 5,000 – 6,000/µm^2^ (or approx. 10,000/µm^3^ in 3D assuming fBALM sectioning capability studied in **Supp. Fig. S6**). In these images (**Fig. 4, 5**) the structural resolution estimate based on a sampling criterion in 2D can be expressed by (Legant et al., 2016):

$R=\sqrt{\left( 2.35\sigma\right)^{2}+ \left[ \frac{2}{\sqrt{d}} \right]^{2}}$ , (1)

where $d$ stands for localisation density. For localisation precision (*σ*) of 20 nm, the structural 2D resolution estimate according to this formula amounts to approximately 53 nm (lower bound) which is in agreement with the smallest structures discernible in our fBALM acquisitions. We note however, that this estimate is somewhat arbitrary; the equation 1 holds true only when assuming a periodic sampling with regular intervals; this is not the case in SMLM as here the structure is sampled by single molecule signals randomly. This necessitates localisation densities which are a factor of $\varphi=5$ to 10 higher than the value indicated by the Nyquist sampling criterion for sampling at fixed intervals (Legant et al., 2016):
 $R_{SMLM}=\sqrt{(2.35{\sigma)}^{2}+\left( \frac{2}{\left( \varphi d_{0} \right)^{\frac{1}{N}}} \right)^{2}}$ , (2)

where $\varphi$ is an oversampling factor, *N* is a dimensionality of the sample, and $d_{0}$ is the density when sampling according to the Nyquist criterion. Understandably, according to the Equation (2), in order to reach the resolution of 53 nm, the localisation density $d$ needs to be at least 5x higher than Nyquist density $d_{0}$. However, $d$ obtainable in our fBALM images is a blindly assessed value that does account neither for the sample sparseness nor for the variation of the signal detection probability along the optical axis. The applicability of Equations (1) and (2) to experimental data has been the subject of an ongoing debate (see e.g. (Gould et al., 2012)). In a previous study, using a 3D microtome serial block face scanning electron microscopy, it has been estimated that chromatin occupies only ~34% in hepatocyte and 58% in endothelial cell nuclei volume (Roquette et al. 2009). In order to obtain a true structural resolution of 53 nm, we would still need an additional oversampling of a factor of 2-3 with respect to the signal density that was obtained in our measurements.

Therefore, taking into account the sample sparseness (50%) and section thickness of 500 nm (**Supp. Fig. S6**) our 3D localisation density *d* within labelled structures amounts to 5,000 SM/µm²/50%/500nm = 20,000 SM/µm^3^. Assuming oversampling factor of ϕ=5 (as recommended in (Legant et al., 2016)) the Nyquist sampling density *d_0_* equals to 4,000 SM/µm^3^. Now, back-calculating the possible structural resolution equivalent using equation 1 and localisation precision of 20 nm the resolution estimate of fBALM corresponds to 134 nm assuming a 3D structure and to 65 nm assuming a 2D structure.

**Supplementary Note 3 - DNA structure fluctuation-assisted Binding Activated Localisation Microscopy (fBALM) – acquisition troubleshooting**

In the DNA phase fluctuation assisted BALM (fBALM), several factors have to be taken into account in order to perform super-resolution imaging of the nuclear DNA. Among most important of them are: (i) DNA dye penetration to the interior of the cell nucleus that can be studied also by investigation of the association kinetics similarly as we did in **Supp. Fig. S1**. Only dyes that penetrate the nucleus very slowly or very rapidly are useful for fBALM of the cell nucleus. The first ones can be delivered to the cell nucleus prior to a measurement for as long as it is required for the DNA to be fully labelled; however, upon acidic conditions they will not be able to leave the cell nucleus rapidly (YOYO-1 is an example of such a dye). The latter ones would penetrate the cell nucleus fast enough even when added to the imaging medium during the BALM measurement. We failed to find such dyes and even relatively small PicoGreen and YO-PRO-1 diffused too slowly). (ii) Intranuclear, unbound dye concentration after slow DNA denaturation can be controlled by an appropriate permeabilisation of the fixed cells; too high detergent concentrations as well as too long detergent treatment may lead to a very rapid loss of the DNA dye in the cell nucleus upon treatment with e.g. acidic conditions. In such circumstances the dye will undergo a relocation to the imaging medium and may contribute to peripheral artefacts (similar to as shown in **Supp. Fig. S1B, Supp. Fig. S9**) as it will re-bind to the most accessible DNA at the nuclear boundary. A similar situation is true for very long SMLM acquisitions (e.g. 100,000 frames): in this case, the last frames contain usually single molecule signals originating from the nuclear periphery. While the intranuclear pool of the YOYO-1 undergoes a progressive photobleaching, the extracellular dye binds to the most accessible DNA at the periphery, effectively not contributing to resolution improvement. If these peripheral effects would not occur, longer measurements would allow us to filter more strongly on the brightness of the localisations and increase both localisation precision and labelling density. (iii) Different cell types may necessitate different permeabilisation protocols as their cell membranes often differ in composition (Spector and Yorek, 1985). (iv) Different cell types may have varying nuclear sizes resulting in a different dye accessibility to the interior. Assuming a spheroidal shape of the cell nucleus, a surface *s* of a nucleus with a radius *r* will scale as *s=4πr^2^ .*This means that a nucleus with a radius twice smaller will have a surface 4 times smaller. The surface (membrane) permeability in this case is crucial for proper super-resolution fBALM imaging and thus needs to be taken into account. (v) The volume of the imaging buffer typically influences the concentration of the freely diffusing DNA dye that underwent detachment from the dsDNA (during for instance. a pH drop), and escaped from the cell nucleus. Hence, by keeping the volume as high as possible one can reduce a threat of peripheral artefacts stemming from re-binding of the dye present in the imaging buffer to the nuclear periphery. In our experiments, we used typically a volume of ~30 µl only, however, it might be increased easily even up to 1000 µl.

**Supplementary Note 4 – Present limitations in fBALM methodology**

A few methodological aspects and potential limitations need to be addressed to properly interpret the data in DNA structure fluctuation assisted BALM presented here.

First, the nuclear DNA is not fully separated into single stranded DNA even if lower pH was used (Pierzyńska-Mach et al., 2016). Hence, we hypothesise that a slight bias might be introduced towards some DNA sequences that are localised by transient single YOYO-1 molecule fluorescence emission. It has been shown previously that binding of YOYO-1 to DNA in denaturing environment containing formamide with temperature gradually raised, leads to local fluorescence signal loss in sequences of lower local melting temperatures. This has been proven useful for coarse-grain DNA sequencing based on fluorescence microscopy (Reisner et al., 2010). Such sequences are typically AT-rich and contain only 2 hydrogen bonds unlike in GC base-pairs that contain 3 hydrogen bonds. We anticipate that in fBALM methodology described here, such AT-rich sequences may be more prone to local phase changes (dsDNA ↔ ssDNA); hence transient binding-unbinding of YOYO-1 might occur there more frequently than elsewhere (e.g. where the DNA dye remains permanently bound to dsDNA and undergoes an irreversible photobleaching during a measurement under high intensity excitation).

Second, an additional important effect on DNA stability that may contribute to some sequence bias of fBALM is DNA base protonation that precedes acid-induced DNA denaturation. This phenomenon is known to proceed differently for GC-pairs and AT-pairs (reviewed in (Löber and Zimmer, 1968; Hermann and Fredericq, 1977).

Third, in the single molecule microscopy method presented here one needs to consider also an effect of dye photoblinking. Here we have not used a reductive-oxidative system for fluorescence stabilisation (Vogelsang et al., 2008) because (i) its common component ascorbic acid, will influence the pH of our well defined system (**Fig. 3B**) which in turn may affect DNA stability and nuclear structure (**Fig. 2**), and (ii) because the photophysics of DNA-bound dyes in the context of radical non-emitting state formation is still vague. Note that in our fBALM approach, YOYO-1 molecules may blink once bound to the DNA, and binding-dissociation is likely a primary but not a sole “bright-“ / “dark-state” switching mechanism in this approach. This may lead to a slight overestimation of binding events in the final analysis, as the same molecule bound to an individual intercalating site might reappear even after hundreds of frames passed. The existence of dark states for YOYO-1 was previously suggested in the presence of MEA (Flors, 2010) and this can be further reinforced by the fact that in our experiment YOYO-1 fluorescence recovered almost completely upon a wash (**Fig. 1A**).

Fourth, another issue to be considered is the fact that as long as the density of signals in fBALM can be assumed to correspond with the “underlying” DNA density, a single fluorescence signal cannot be attributed to a single YOYO-1 molecule event. We expect that local structure instabilities leading to local dsDNA formation may attract more than one dye molecule at once (YOYO-1 is known to bind even every 3.2 +/- 0.6 bp (Günther et al., 2010)). Furthermore, we cannot exclude that a single dye molecule is detected several times. Additionally, the same DNA binding site can be sampled more than once as the DNA conformational changes in this pH-range are reversible (Zimmer et al., 1968). These effects have to be taken into consideration when drawing conclusions from the detected density of signals.

Lastly, even though the mechanism of fluorescence photoswitching in fBALM is likely dominated by DNA conformational fluctuations, the effects on DNA-binding dye structure may also play a significant role. For instance, YOYO-1 has 4 protonation sites that while partially protonated in various combinations may influence its photophysical features (in a similar manner as in Hoechst dyes (Żurek-Biesiada et al., 2013; 2014)) as well as its DNA-binding strength. The latter putative effect could likely stem from an alteration to the dye’s conformation or different electrostatic interaction with the DNA.

**References to supplementary materials:**

Alonso, A., Almendral, M.J., Curto, Y., Criado, J.J., Rodriguez, E., Manzano, J.L., 2006. Determination of the DNA-binding characteristics of ethidium bromide, proflavine, and cisplatin by flow injection analysis: Usefulness in studies on antitumor drugs. Anal. Biochem. 355, 157–164. doi:10.1016/j.ab.2006.06.004

Backer, A.S., Lee, M.Y., Moerner, W.E., 2016. Enhanced DNA imaging using super-resolution microscopy and simultaneous single-molecule orientation measurements. Optica 3, 659. doi:10.1364/OPTICA.3.000659

Baddeley, D., Cannell, M.B., Soeller, C., 2010. Visualization of localization microscopy data. Microsc. Microanal. 16, 64–72. doi:10.1017/S143192760999122X

Bancaud, A., Huet, S., Daigle, N., Mozziconacci, J., Beaudouin, J., Ellenberg, J., 2009. Molecular crowding affects diffusion and binding of nuclear proteins in heterochromatin and reveals the fractal organization of chromatin. EMBO J. 28, 3785–98. doi:10.1038/emboj.2009.340

Banterle, N., Bui, K.H., Lemke, E.A., Beck, M., 2013. Fourier ring correlation as a resolution criterion for super-resolution microscopy. J. Struct. Biol. 183, 363–7. doi:10.1016/j.jsb.2013.05.004

Benvin, A.L., Creeger, Y., Fisher, G.W., Ballou, B., Alan, S., Armitage, B. a, 2008. NIH Public Access 129, 2025–2034. doi:10.1021/ja066354t.Fluorescent

Burgert, A., Letschert, S., Doose, S., Sauer, M., 2015. Artifacts in single-molecule localization microscopy. Histochem. Cell Biol. 144, 123–31. doi:10.1007/s00418-015-1340-4

Chen, J., Bremauntz, A., Kisley, L., Shuang, B., Landes, C.F., 2013. Super-resolution mbPAINT for optical localization of single-stranded DNA. ACS Appl. Mater. Interfaces 5, 9338–9343. doi:10.1021/am403984k

Claycomb, W.C., Lanson, N.A., Stallworth, B.S., Egeland, D.B., Delcarpio, J.B., Bahinski, A., Izzo, N.J., 1998. HL-1 cells: a cardiac muscle cell line that contracts and retains phenotypic characteristics of the adult cardiomyocyte. Proc. Natl. Acad. Sci. U. S. A. 95, 2979–2984. doi:10.1073/pnas.95.6.2979

Cosa, G., Focsaneanu, K.S., McLean, J.R., McNamee, J.P., Scaiano, J.C., 2001. Photophysical properties of fluorescent DNA-dyes bound to single- and double-stranded DNA in aqueous buffered solution. Photochem. Photobiol. 73, 585–99.

Cremer, T., Cremer, M., Hübner, B., Strickfaden, H., Smeets, D., Popken, J., Sterr, M., Markaki, Y., Rippe, K., Cremer, C., 2015. The 4D nucleome: Evidence for a dynamic nuclear landscape based on co-aligned active and inactive nuclear compartments. FEBS Lett. 589, 2931–2943. doi:10.1016/j.febslet.2015.05.037

Dragan, A.I., Casas-Finet, J.R., Bishop, E.S., Strouse, R.J., Schenerman, M.A., Geddes, C.D., 2010. Characterization of PicoGreen interaction with dsDNA and the origin of its fluorescence enhancement upon binding. Biophys. J. 99, 3010–3019. doi:10.1016/j.bpj.2010.09.012

Dragan, A.I., Pavlovic, R., McGivney, J.B., Casas-Finet, J.R., Bishop, E.S., Strouse, R.J., Schenerman, M.A., Geddes, C.D., 2012. SYBR Green I: Fluorescence properties and interaction with DNA. J. Fluoresc. 22, 1189–1199. doi:10.1007/s10895-012-1059-8

Drobyshev, A.L., Zasedatelev, A.S., Yershov, G.M., Mirzabekov, A.D., 1999. Massive parallel analysis of DNA-Hoechst 33258 binding specificity with a generic oligodeoxyribonucleotide microchip. Nucleic Acids Res. 27, 4100–4105. doi:10.1093/nar/27.20.4100

Flors, C., 2010. Photoswitching of monomeric and dimeric DNA-intercalating cyanine dyes for super-resolution microscopy applications. Photochem. Photobiol. Sci. 9, 643–8. doi:10.1039/b9pp00119k

Glazer, A.N., Rye, H.S., 1992. Stable dye-DNA intercalation complexes as reagents for high-sensitivity fluorescence detection. Nature 359, 859–61. doi:10.1038/359859a0

Gould, T.J., Hess, S.T., Bewersdorf, J., 2012. Optical Nanoscopy: From Acquisition to Analysis. Annu. Rev. Biomed. Eng. 14, 231–254. doi:10.1146/annurev-bioeng-071811-150025

Gruell, F., Kirchgessner, M., Kaufmann, R., Hausmann, M., Kebschull, U., 2011. Accelerating Image Analysis for Localization Microscopy with FPGAs, in: 2011 21st International Conference on Field Programmable Logic and Applications. IEEE, pp. 1–5. doi:10.1109/FPL.2011.11

Günther, K., Mertig, M., Seidel, R., 2010. Mechanical and structural properties of YOYO-1 complexed DNA. Nucleic Acids Res. 38, 6526–6532. doi:10.1093/nar/gkq434

Hermann, P., Fredericq, E., 1977. The role of the AT pairs in the acid denaturation of DNA. Nucleic Acids Res. 4, 2939–2947. doi:10.1093/nar/4.8.2939

Himo, F., Lovell, T., Hilgraf, R., Rostovtsev, V. V, Noodleman, L., Sharpless, K.B., Fokin, V. V, 2005. Copper(I)-catalyzed synthesis of azoles. DFT study predicts unprecedented reactivity and intermediates. J. Am. Chem. Soc. 127, 210–216. doi:10.1021/ja0471525

Joseph, M.J., Taylor, J.C., McGown, L.B., Pitner, J.B., Linn, C.P., 1998. Spectroscopic studies of YO and YOYO fluorescent dyes in a thrombin-binding DNA ligand. Biospectroscopy 2, 173–183. doi:10.1002/(SICI)1520-6343(1996)2:3<173::AID-BSPY4>3.0.CO;2-9

Kirmes, I., Szczurek, A., Prakash, K., Charapitsa, I., Heiser, C., Musheev, M., Schock, F., Fornalczyk, K., Ma, D., Birk, U., Cremer, C., Reid, G., 2015. A transient ischemic environment induces reversible compaction of chromatin. Genome Biol. 16, 246. doi:10.1186/s13059-015-0802-2

Legant, W.R., Shao, L., Grimm, J.B., Brown, T.A., Milkie, D.E., Avants, B.B., Lavis, L.D., Betzig, E., 2016. High-density three-dimensional localization microscopy across large volumes. Nat. Methods 1–9. doi:10.1038/nmeth.3797

Löber, G., Zimmer, C.H., 1968. pH induced changes in the UV-absorption band of deoxyribonucleic acid. Biochem. Biophys. Res. Commun. 31, 641–645. doi:10.1016/0006-291X(68)90527-5

Manzini, G., Xodo, L., Barcellona, M.L., Quadrifoglio, F., 1985. Interaction of DAPI with double-stranded ribonucleic acids. Nucleic Acids Res. 13, 8955–8967. doi:10.1093/nar/13.24.8955

Mortensen, K.I., Churchman, L.S., Spudich, J.A., Flyvbjerg, H., 2010. Optimized localization analysis for single-molecule tracking and super-resolution microscopy. Nat. Methods 7, 377–81. doi:10.1038/nmeth.1447

Nieuwenhuizen, R.P.J., Lidke, K. a, Bates, M., Puig, D.L., Grünwald, D., Stallinga, S., Rieger, B., 2013. Measuring image resolution in optical nanoscopy. Nat. Methods 10, 557–62. doi:10.1038/nmeth.2448

Njoh, K.L., Patterson, L.H., Zloh, M., Wiltshire, M., Fisher, J., Chappell, S., Ameer-Beg, S., Bai, Y., Matthews, D., Errington, R.J., Smith, P.J., 2006. Spectral analysis of the DNA targeting bisalkylaminoanthraquinone DRAQ5 in intact living cells. Cytom. Part A 69, 805–814. doi:10.1002/cyto.a.20308

Pierzyńska-Mach, A., Szczurek, A., Cella Zanacchi, F., Pennacchietti, F., Drukała, J., Diaspro, A., Cremer, C., Darzynkiewicz, Z., Dobrucki, J.W., 2016. Subnuclear localization, rates and effectiveness of UVC-induced unscheduled DNA synthesis visualized by fluorescence widefield, confocal and super-resolution microscopy. Cell Cycle 15, 1156–67. doi:10.1080/15384101.2016.1158377

Reisner, W., Larsen, N.B., Silahtaroglu, A., Kristensen, A., Tommerup, N., Tegenfeldt, J.O., Flyvbjerg, H., 2010. Single-molecule denaturation mapping of DNA in nanofluidic channels. Proc. Natl. Acad. Sci. 107, 13294–13299. doi:10.1073/pnas.1007081107

Ricci, M.A., Manzo, C., García-Parajo, M.F., Lakadamyali, M., Cosma, M.P., 2015. Chromatin Fibers Are Formed by Heterogeneous Groups of Nucleosomes In Vivo. Cell 160, 1145–1158. doi:10.1016/j.cell.2015.01.054

Ries, J., Kaplan, C., Platonova, E., Eghlidi, H., Ewers, H., 2012. A simple, versatile method for GFP-based super-resolution microscopy via nanobodies. Nat. Methods 9, 582–584. doi:10.1038/nmeth.1991

Righolt, C.H., Guffei, A., Knecht, H., Young, I.T., Stallinga, S., van Vliet, L.J., Mai, S., 2014. Differences in nuclear DNA organization between lymphocytes, Hodgkin and Reed-Sternberg cells revealed by structured illumination microscopy. J. Cell. Biochem. 115, 1441–8. doi:10.1002/jcb.24800

Rye, H.S., Yue, S., Wemmer, D.E., Quesada, M. a, Haugland, R.P., Mathies, R. a, Giazer, A.N., 1992. bis-intercalating asymmetric cyanine dyes : properties and applications CO-. Nucleic Acids Res. 20, 2803–2812.

Schermelleh, L., Carlton, P.M., Haase, S., Shao, L., Winoto, L., Kner, P., Burke, B., Cardoso, M.C., Agard, D. a, Gustafsson, M.G.L., Leonhardt, H., Sedat, J.W., 2008. Subdiffraction multicolor imaging of the nuclear periphery with 3D structured illumination microscopy. Science 320, 1332–6. doi:10.1126/science.1156947

Schoen, I., Ries, J., Klotzsch, E., Ewers, H., Vogel, V., 2011. Binding-Activated Localization Microscopy of DNA Structures. Nano Lett. 11, 4008–4011. doi:10.1021/nl2025954

Smith, P.J., Blunt, N., Wiltshire, M., Hoy, T., Teesdale-Spittle, P., Craven, M.R., Watson, J. V., Brad Amos, W., Errington, R.J., Patterson, L.H., 2000. Characteristics of a novel deep red/infrared fluorescent cell-permeant DNA probe, DRAQ5, in intact human cells analyzed by flow cytometry, confocal and multiphoton microscopy. Cytometry 40, 280–291. doi:10.1002/1097-0320(20000801)40:4<280::AID-CYTO4>3.0.CO;2-7

Sorokin, V.A., Gladchenko, G.O., Valeev, V.A., 1986. DNA protonation at low ionic strength of solution. Die Makromol. Chemie 187, 1053–1063. doi:10.1002/macp.1986.021870502

Spector, A.A., Yorek, M.A., 1985. Membrane lipid composition and cellular function. J. Lipid Res. 26, 1015–1035. doi:3906008

Szczurek, A.T., Prakash, K., Lee, H.-K., Żurek-Biesiada, D.J., Best, G., Hagmann, M., Dobrucki, J.W., Cremer, C., Birk, U., 2014. Single molecule localization microscopy of the distribution of chromatin using Hoechst and DAPI fluorescent probes. Nucleus 5, 331–340. doi:10.4161/nucl.29564

Traganos, F., Darzynkiewicz, Z., Sharpless, T., Melamed, M.R., 1975. Denaturation of deoxyribonucleic acid in situ effect of formaldehyde. J. Histochem. Cytochem. 23, 431–8.

Vardevanyan, P.O., Antonyan, a P., Parsadanyan, M. a, Davtyan, H.G., Karapetyan, a T., 2003. The binding of ethidium bromide with DNA: interaction with single- and double-stranded structures. Exp. Mol. Med. 35, 527–533. doi:10.1038/emm.2003.68

Vaughan, J.C., Jia, S., Zhuang, X., 2012. Ultrabright photoactivatable fluorophores created by reductive caging. Nat. Methods 9, 1181–4. doi:10.1038/nmeth.2214

Verdolino, V., Cammi, R., Munk, B.H., Schlegel, H.B., 2008. Calculation of p K a Values of Nucleobases and the Guanine Oxidation Products Guanidinohydantoin and Spiroiminodihydantoin using Density Functional Theory and a Polarizable Continuum Model. J. Phys. Chem. B 112, 16860–16873. doi:10.1021/jp8068877

Vogelsang, J., Kasper, R., Steinhauer, C., Person, B., Heilemann, M., Sauer, M., Tinnefeld, P., 2008. A reducing and oxidizing system minimizes photobleaching and blinking of fluorescent dyes. Angew. Chemie - Int. Ed. 47, 5465–5469. doi:10.1002/anie.200801518

Wang, Q., Chan, T.R., Hilgraf, R., Fokin, V. V, Sharpless, K.B., Finn, M.G., 2003. Bioconjugation by Copper(I)-Catalyzed Azide-Alkyne [3 + 2] Cycloaddition. J. Am. Chem. Soc. 125, 3192–3193. doi:10.1021/ja021381e

Zessin, P.J.M., Finan, K., Heilemann, M., 2012. Super-resolution fluorescence imaging of chromosomal DNA. J. Struct. Biol. 177, 344–8. doi:10.1016/j.jsb.2011.12.015

Zhao, H., Halicka, H.D., Li, J., Biela, E., Berniak, K., Dobrucki, J., Darzynkiewicz, Z., 2013. DNA damage signaling, impairment of cell cycle progression, and apoptosis triggered by 5-ethynyl-2’-deoxyuridine incorporated into DNA. Cytometry. A 83, 979–88. doi:10.1002/cyto.a.22396

Zimmer, C., Luck, G., Venner, H., Fric, J., 1968. Studies on the conformation of protonated DNA. Biopolymers 6, 563–574. doi:10.1002/bip.1968.360060410

Żurek-Biesiada, D., Kędracka-Krok, S., Dobrucki, J.W., 2013. UV-activated conversion of Hoechst 33258, DAPI, and Vybrant DyeCycle fluorescent dyes into blue-excited, green-emitting protonated forms. Cytometry. A 83, 441–51. doi:10.1002/cyto.a.22260

Żurek-Biesiada, D., Waligórski, P., Dobrucki, J.W., 2014. UV-induced Spectral Shift and Protonation of DNA Fluorescent Dye Hoechst 33258. J. Fluoresc. doi:10.1007/s10895-014-1468-y
